# Supplementary material for: Jointly representing long-range genetic similarity and spatially heterogeneous isolation-by-distance
Source: PLoS Genet. 2025 Sep 16;21(9):e1011612. doi: 10.1371/journal.pgen.1011612 (PMC12453258; doi:10.1371/journal.pgen.1011612)
Supplement: S1 Text — (PDF) [file pgen.1011612.s001.pdf]

S1 Features of empirical data

S1.1 North American gray wolves (*Canis lupus*)

The data set of 111 individuals sampled across North America was originally collected in Schweizer et al. (2016) and used as an example data set in `FEEMS`. The SNPs were pruned for a 5% minor allele frequency cutoff and a 10% missingness rate, resulting in a total of 17,729 SNPs. Since `FEEMSmix` is built on top of `FEEMS`, we used the same dense grid chosen in the original analysis to fit the baseline model. This grid had a cell area of approx. 6,200 sq. km. and a cell spacing of approx. 110 km. As a result of this grid choice, we see that the 111 individuals get assigned to 94 demes across the species' continental range. Both `TreeMix` and `SpaceMix` were run on this sampled deme level to enable a close comparison with the results from `FEEMSmix`.

S1.2 Afro-Eurasian panel of humans (*Homo sapiens*)

We start with the data set of 4,302 individuals curated in Peter et al. (2020) for our analysis. From this data, we subset to those with public sharing permissions and are left with 4,070 individuals. This final data set consists of 19,954 SNPs from 319 distinct sampling locations across Afro-Eurasia. For this analysis, we use a grid with cell area of approx. 25,000 sq. km. and a cell spacing of approx. 220 km., resulting in a high sampling resolution with a total of 290 sampled demes across the grid.

References

Alexander, D. H., J. Novembre, and K. Lange (2009). Fast model-based estimation of ancestry in unrelated individuals. *Genome Research 19*(9), 1655–1664.

Battey, C. J., P. L. Ralph, and A. D. Kern (2020). Predicting geographic location from genetic variation with deep neural networks. *eLife 9*, e54507.

Bradburd, G. S., P. L. Ralph, and G. M. Coop (2016). A spatial framework for understanding population structure and admixture. *PLoS Genetics 12*(1), e1005703.

Marcus, J., W. Ha, R. F. Barber, and J. Novembre (2021). Fast and flexible estimation of effective migration surfaces. *eLife 10*, e61927.

Met Office (2010 - 2015). *Cartopy: a cartographic python library with a Matplotlib interface.* Exeter, Devon.

Patterson, N., P. Moorjani, Y. Luo, S. Mallick, N. Rohland, Y. Zhan, T. Genschoreck, T. Webster, and D. Reich (2012). Ancient admixture in human history. *Genetics 192*(3), 1065–1093.

Peter, B. M., D. Petkova, and J. Novembre (2020). Genetic landscapes reveal how human genetic diversity aligns with geography. *Molecular Biology and Evolution 37*(4), 943–951.

Pickrell, J. and J. Pritchard (2012). Inference of population splits and mixtures from genome-wide allele frequency data. *PLoS Genet 8*(11), e1002967.

Schweizer, R. M., B. M. Vonholdt, R. Harrigan, J. C. Knowles, M. Musiani, D. Coltman, J. Novembre, and R. K. Wayne (2016). Genetic subdivision and candidate genes under selection in North American grey wolves. *Molecular Ecology 25*(1), 380–402.

Shastry, V., M. Musiani, and J. Novembre (2025). Data from: Jointly representing long-range genetic similarity and spatially heterogeneous isolation-by-distance.
