# Supplementary material for: Jointly representing long-range genetic similarity and spatially heterogeneous isolation-by-distance
Source: PLoS Genet. 2025 Sep 16;21(9):e1011612. doi: 10.1371/journal.pgen.1011612 (PMC12453258; doi:10.1371/journal.pgen.1011612)
Supplement: S2 Text — (PDF) [file pgen.1011612.s002.pdf]

S2 Results from North American gray wolf samples of Schweizer et al. (2016)

S2.1 Using SpaceMix

`SpaceMix` (Bradburd et al. 2016) was run in two modes: "estimating geogenetic and admixture source locations" and "estimating admixture source locations". As basic preprocessing and to ensure a like-to-like comparison, we clumped individuals into demes specified by the grid used by `FEEMS` and passed as input to `SpaceMix` the sample allele frequency matrix and the number of samples per deme.

In the first mode, the chain was initially burned in for 100,000 steps and then run for a total of 5,000,000 steps with a sampling every 2,000 steps to decrease autocorrelation. This produced 2,500 samples from the posterior distribution. Under this scenario, `SpaceMix` captures the broad geographic patterns in the data well (similar to PCA in Fig S23B), but only implicates a single deme (*737*) with any significant amount of admixture ($> 1\%$, Fig S25). A probable reason for this is that the residuals from other putative admixed sources are modeled away by their 'geogenetic' locations, so a long-range admixture event does not need to be evoked to fit the data (for example, see the locations of demes *621* and *1206*). However, the placement of the other putatively admixed demes (found by both `ADMIXTURE` and `FEEMSmix`) followed their locations in principal components space quite closely.

In the second mode, the chain was initially burned in for 500,000 steps and then run for a total of 35,000,000 steps with a sampling every 10,000 steps. This produced 3,500 samples from the posterior distribution. This mode is similar in idea to `FEEMSmix`, and we found largely overlapping patterns between the two methods (see Fig S26). For instance, six demes with stronger signals of admixture (median admixture proportion $> 5\%$) were implicated in long-range events, and five were in common with `FEEMSmix`: *980, 1206, 621, 180, 207*. Here, the likely location of the sources were very similar in range to locations estimated by `FEEMSmix`, with the median admixture proportions being much lower than the MLE $\hat{c}$ from `FEEMSmix`, with varying degrees of similarity to the values obtained from the previous `ADMIXTURE` analysis.

Finally, convergence for both modes was verified by observing trace plots, acceptance rates and the joint marginal distribution of samples across the different stages of the MCMC. Both modes produced excellent fits to the observed sample covariances with $R^2 > 0.9$ (see Fig S24), indicating that `SpaceMix` does indeed explain the underlying spatial genetic variation in the data.

S2.2 Using TreeMix

`TreeMix` (Pickrell and Pritchard 2012) was run on the allele count matrix for the same set of SNPs across the 94 sampled demes for $m = 1$ to $m = 20$ migration edges using default parameters. We found no significant increase in the log-likelihood of the model on adding edges beyond $m = 15$, so we use the resulting topology with 15 migration edges for our interpretations in the main text (see Fig S11A).

In general, TreeMix also captures the broad geographic and ecotypic patterns present in the wolves well (see Fig S11A). A few notable exceptions in the tree structure were the placement of deme *621* next to the WestForest populations and the location of deme *1206* as being an outgroup to the rest of the populations. With regard to the migration edges, 14 our of 15 edges were between Arctic and HighArctic populations and did not replicate patterns found by SpaceMix or FEEMSmix. But, interestingly, FEEMSmix does produce a similar pattern when run under a baseline FEEMS fit with a *single, fixed variance* across all demes (see Fig S5). These Arctic and HighArctic populations also showed the highest amounts of drift under the TreeMix model and since the deme-specific variance parameter in FEEMS captures a quantity proportional to the effective population size, we believe that by estimating this quantity, we model away any such residuals that TreeMix picks up (hence, just a single outlier from this region with under the default model of a *deme-specific variance*). The singular edge in TreeMix that is *not* between Arctic and HighArctic populations is actually from the base of several different populations to the branch leading down to the AtlanticForest populations of which *980* (largest outlier in FEEMSmix) is a part of. In short, TreeMix only seems to model this largest signal of long-range gene flow, but misses on the more subtle signals captured by ADMIXTURE, FEEMSmix and SpaceMix.

S2.3 Using FEEMSmix

We identified $K = 10$ long-range edges (LREs) upon *iterative* fitting of the wolves data with 9 unique recipient demes (see Fig 3A). To better understand the inclusion of these edges in the results, we investigated the sample meta-data. There, we found that 2 out of the 9 recipient demes contained samples with recording errors, and 3 out of the 9 recipient demes contained samples that were registered to questionable locations. During our investigation, we also found evidence of one more sample (*189*) as being misrecorded, and this was implicated as the 24th LRE in our analysis (see Fig S28). We find that the inferred locations are in the same general direction as the true locations (though, in some cases, off by a few hundred kilometers, see Fig 4A). Deme *621* is implicated in a SpaceMix analysis that places its inferred source in the same direction as FEEMSmix, but much farther west and north than its true location (see Fig S26). Deme *189* is implicated as an outlier in TreeMix as well.

For the samples that were reported as coming from questionable locations, we found that sample *815* was assigned to the centroid of a national park area (indicating a proxy for the region for where it was found). With this sample, we found that the inferred locations in FEEMSmix are proximal to their recorded locations, which is a loose indicator that this wolf may habit this general area but may not actually be from this specific location (and, as a result, are found to be an outlier by the method). For the other two samples (*180, 207*), we found in the sample meta-data that both these samples were registered in the town of Invuik (black plus sign in Fig 4B) in the Northwest Territories of Canada. This is the only town in the region, so we infer that these wolves were most likely hunted somewhere else and brought to this town for processing (and, eventually, registered). FEEMSmix infers the source locations for this sample to be southeast of this town. This is in the same general direction as the source inferred by SpaceMix (see Fig S26). A supporting result is that in TreeMix's inferred tree (which is fit without any spatial information) the samples closest

to the FEEMSmix MLE source deme and those from the destination deme are inferred to be sister populations.

We note that SpaceMix also implicates the 2 out of 3 samples that were identified as being recorded at questionable locations (demes *180, 207*) and 1 out of 3 samples that were results of recording errors (deme *621*), whereas TreeMix only finds one 1 out of 3 samples that were results of recording errors (deme *189*).

Out of the remaining 4 samples, 3 samples (demes *980, 1206, 187*) show admixed ancestry in an ADMIXTURE analysis (Fig 4B), of which 2 out of 3 (demes *980, 1206*) are also implicated in a SpaceMix analysis (Fig S25). Out of these two outliers, deme *980* is found to be the source of a long-range migration event in TreeMix, though deme *1206* is not. Based on the inference of partial admixture memberships to multiple ancestries to deme *1206* and its basal outgroup position in the tree (and its location as an isolated sample on the westernmost island off the coast of Alaska), we hypothesize that this sample is likely a descendant of an ancestral wolf population that spread eastwards and gave rise to the populations that were eventually sampled from the central and eastern parts of the continental range. (This represents a model mis-specification under both FEEMSmix and SpaceMix.) Finally, the one deme that is a somewhat puzzling inclusion in FEEMSmix is deme *834* which doesn't show multiple memberships in an ADMIXTURE analysis, but still requires two LREs with different sources to account for the remaining residual under the model.

Finally, we present a reanalysis of the wolf samples with the locations corrected and the ambiguous samples removed in the section below.

S2.3.1 Re-analysis of the corrected wolf samples with FEEMSmix

After the correction, we are now left with 108 wolf samples spread over 89 unique nodes in the graph, where $\sim 90\%$ of nodes contained just a single sample (provided in Shastry et al. 2025). The estimated migration surface over these samples (Fig S28A) is very similar to the migration surface estimated in the main text. This baseline fit also provides an excellent fit to the corrected data $R^2 \approx 0.96$. We run the default *iterative* approach with $K = 10$ and find 10 unique recipient demes (see Fig S28A), all showing $L_r > 10$ under our framework.

Out of these 10 demes, we found that 3 (demes *980, 1206, 187*) belong to the outliers from our original analysis in the main text. For all of these demes, the inferred source location and fraction are found to be similar to the estimates from the original analysis. Out of the remaining 7 demes, we found that 2 demes contained reassigned samples from our original analysis (demes *336* and *213* were reassigned demes *585* and *189* from the original analysis, respectively). Interestingly, the LRE to re-assigned deme *189* (deme *213*) now mirrors the migration arrow to this deme from TreeMix. This could indicate that the original location of this sample was initially wrong, but once this was corrected, we see the 'true' signal of long-range genetic similarity. Since TreeMix does not use any spatial information, the results from that method was originally unbiased to this misreporting. We see a similar (albeit slightly weaker) signal for the re-assigned deme *585*, which now shows a source area geographically close to demes *690,822,719,626* and in the Hudson Bay, reflecting its position in the tree inferred by TreeMix. Of the remaining 5 LREs, all reflect directions inferred in TreeMix.

References

Alexander, D. H., J. Novembre, and K. Lange (2009). Fast model-based estimation of ancestry in unrelated individuals. *Genome Research 19*(9), 1655–1664.

Battey, C. J., P. L. Ralph, and A. D. Kern (2020). Predicting geographic location from genetic variation with deep neural networks. *eLife 9*, e54507.

Bradburd, G. S., P. L. Ralph, and G. M. Coop (2016). A spatial framework for understanding population structure and admixture. *PLoS Genetics 12*(1), e1005703.

Marcus, J., W. Ha, R. F. Barber, and J. Novembre (2021). Fast and flexible estimation of effective migration surfaces. *eLife 10*, e61927.

Met Office (2010 - 2015). *Cartopy: a cartographic python library with a Matplotlib interface.* Exeter, Devon.

Patterson, N., P. Moorjani, Y. Luo, S. Mallick, N. Rohland, Y. Zhan, T. Genschoreck, T. Webster, and D. Reich (2012). Ancient admixture in human history. *Genetics 192*(3), 1065–1093.

Peter, B. M., D. Petkova, and J. Novembre (2020). Genetic landscapes reveal how human genetic diversity aligns with geography. *Molecular Biology and Evolution 37*(4), 943–951.

Pickrell, J. and J. Pritchard (2012). Inference of population splits and mixtures from genome-wide allele frequency data. *PLoS Genet 8*(11), e1002967.

Schweizer, R. M., B. M. Vonholdt, R. Harrigan, J. C. Knowles, M. Musiani, D. Coltman, J. Novembre, and R. K. Wayne (2016). Genetic subdivision and candidate genes under selection in North American grey wolves. *Molecular Ecology 25*(1), 380–402.

Shastry, V., M. Musiani, and J. Novembre (2025). Data from: Jointly representing long-range genetic similarity and spatially heterogeneous isolation-by-distance.
