# Supplementary figures and images for: Jointly representing long-range genetic similarity and spatially heterogeneous isolation-by-distance
Source: PLoS Genet. 2025 Sep 16;21(9):e1011612. doi: 10.1371/journal.pgen.1011612 (PMC12453258; doi:10.1371/journal.pgen.1011612)

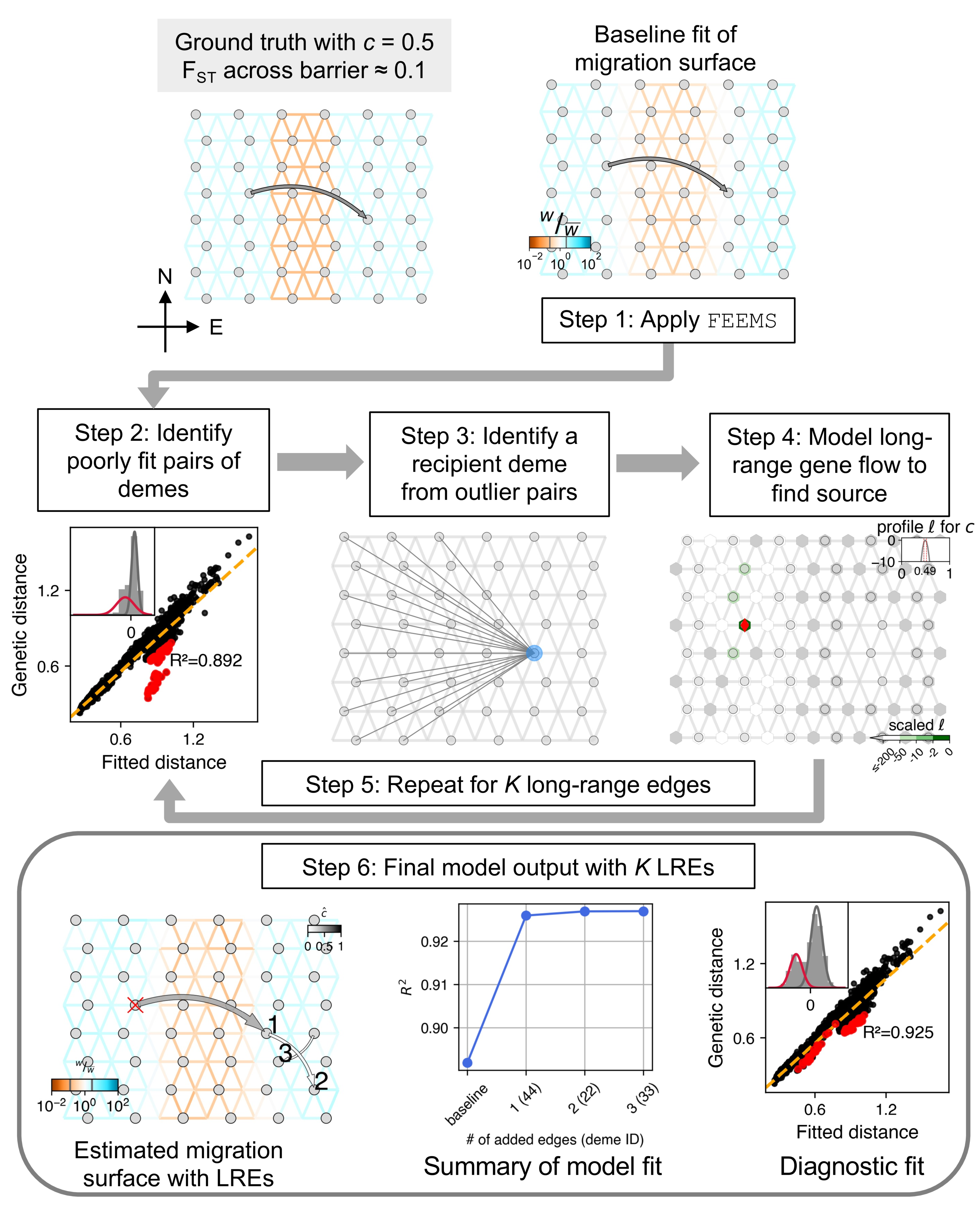

Supplement: S2 Fig — With dense sampling, we see that the baseline FEEMS fit works well to capture the central barrier in the grid, with FEEMSmix also accurately estimating the source and strength of the long-range event. But we also see that adding too many edges can lead to overfitting with a decrease in model R2 with K = 3 LREs. (TIF) [file pgen.1011612.s004.tif]

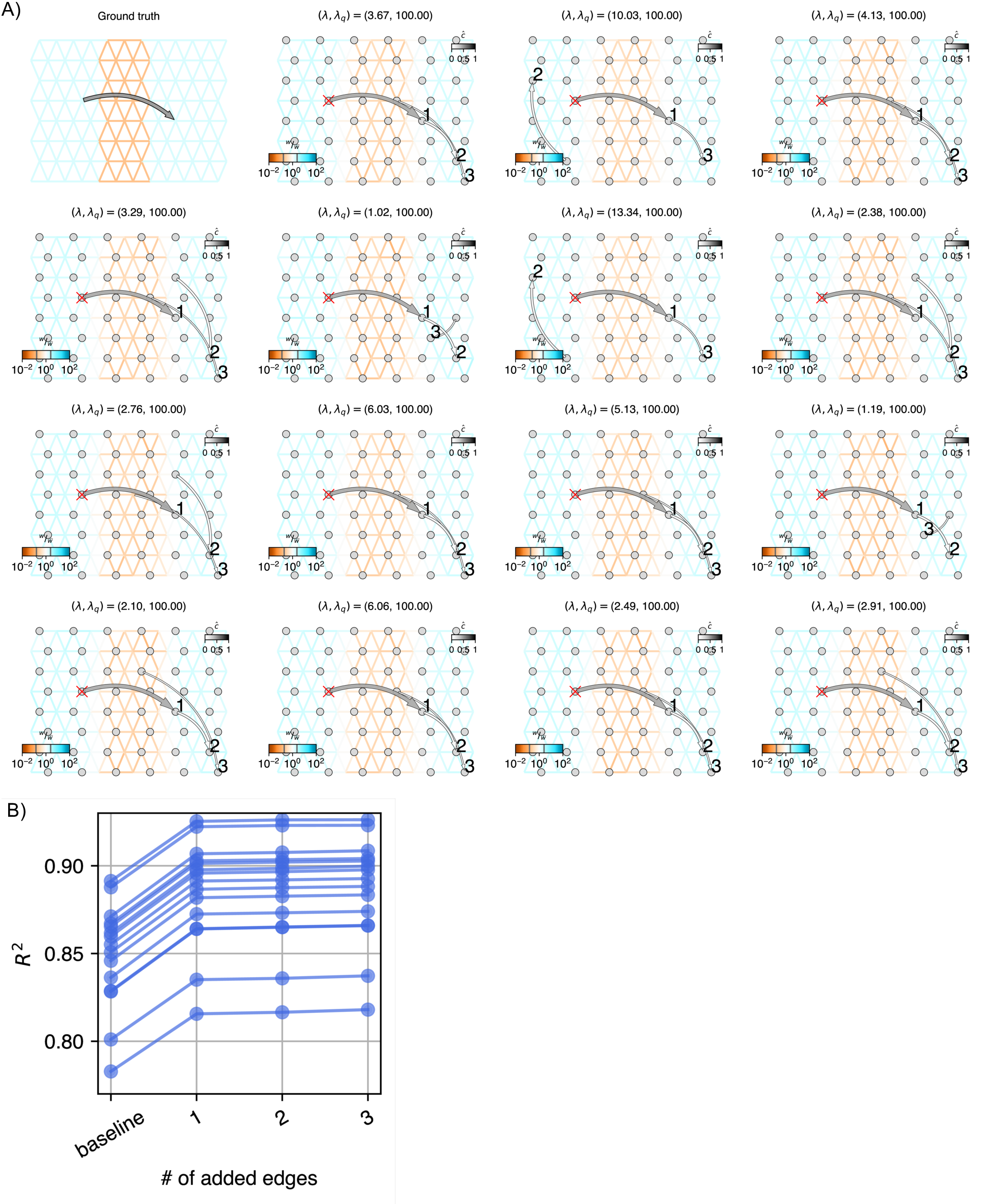

Supplement: S3 Fig — In A), we observe that the true destination deme is always implicated as the first LRE in each replicate. This is true over all 50 simulation replicates. In B), we see a systematic increase in model R2 with the first (true) LRE and a subsequent plateau with more added LREs. In general, over 50 simulation replicates, we find that Lr > 10 in 95%,C95=[86%,100%] of replicates for K = 5 edges indicating that a mixture model of two Normals best fits the residuals with this sampling strategy (see S2 Fig). (TIF) [file pgen.1011612.s005.tif]

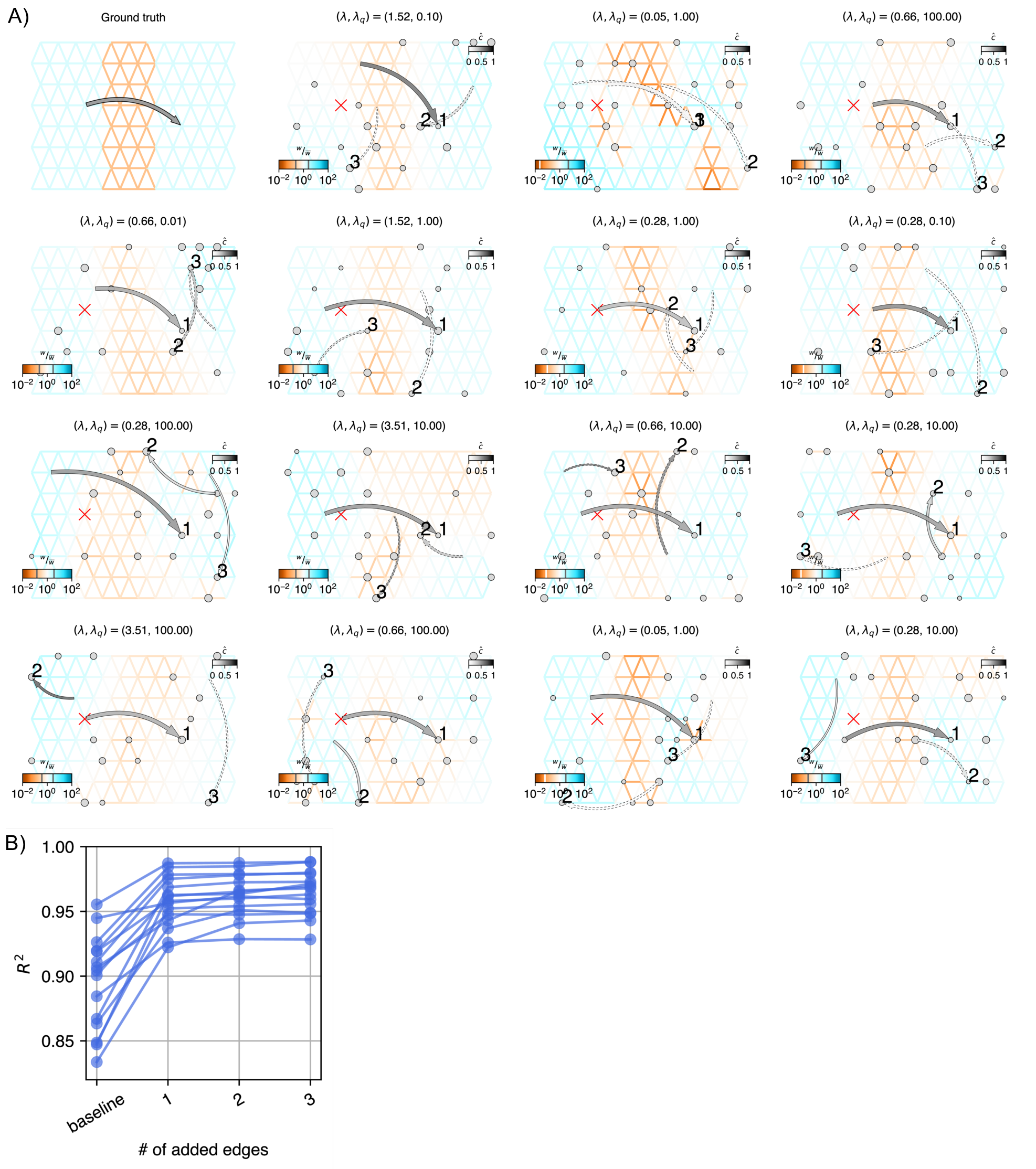

Supplement: S4 Fig — In A), we observe that the true destination deme is implicated as the first LRE in each replicate, while the other LREs are random with respect to the geographical source. In B), we see a systematic increase in model R2 with the first (true) LRE and a subsequent plateau with more added LREs. In general, over 50 simulation replicates, we find that the first LRE always captures the direction and strength of the simulated event even with such sparse sampling. We find that in 94%,C95=[83%,99%] of all simulation replicates the first edge has Lr > 10, while only 30%,C95=[17%,44%] of the simulation replicates show Lr > 10 for the second LRE, dropping down to 5%,[0%,14%] for the third LRE. (TIF) [file pgen.1011612.s006.tif]

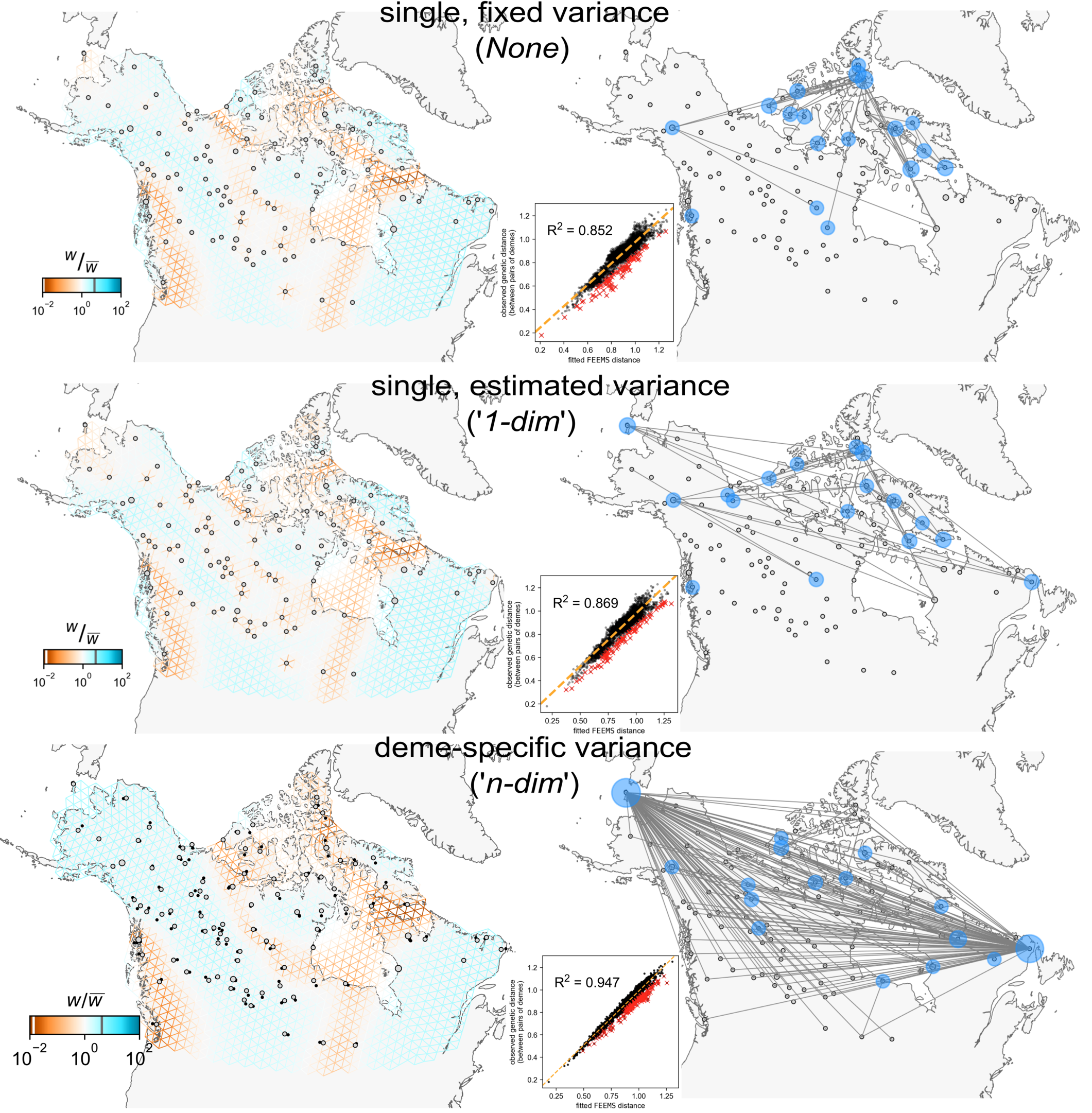

Supplement: S5 Fig — None refers to a single variance parameter that is fixed at a value estimated by a model assuming a single weight across the entire grid [default in 10, comes from an initialization step before optimizing the weights, ∼2.1s], ‘1-dim’ refers to a single, estimated variance parameter that is jointly estimated with all the other weights in the graph (∼13.5s), and ‘n-dim’ refers to estimating a variance parameter for each sampled deme (default in FEEMSmix, ∼11.3s). We observe that we obtain better fits with increasing number of parameters in the framework (based on R2) though with increasing runtime. However, visually, all three methods pick up the major barriers and corridors in the data set. But, an important point to note here is how the pinwheel-like patterns around sampled demes disappear with the estimation of deme-specific variance parameters. Also, the outlier demes implicated in the fits change on a gradient between these modes: with Arctic and HighArctic demes being 75% of outliers in None to just 20% in ‘n-dim’. The base map is drawn using shape files generated by Cartopy (with the base layer available at https://www.naturalearthdata.com/download/50m/physical/ne_50m_land.zip, [36]). (TIF) [file pgen.1011612.s007.tif]

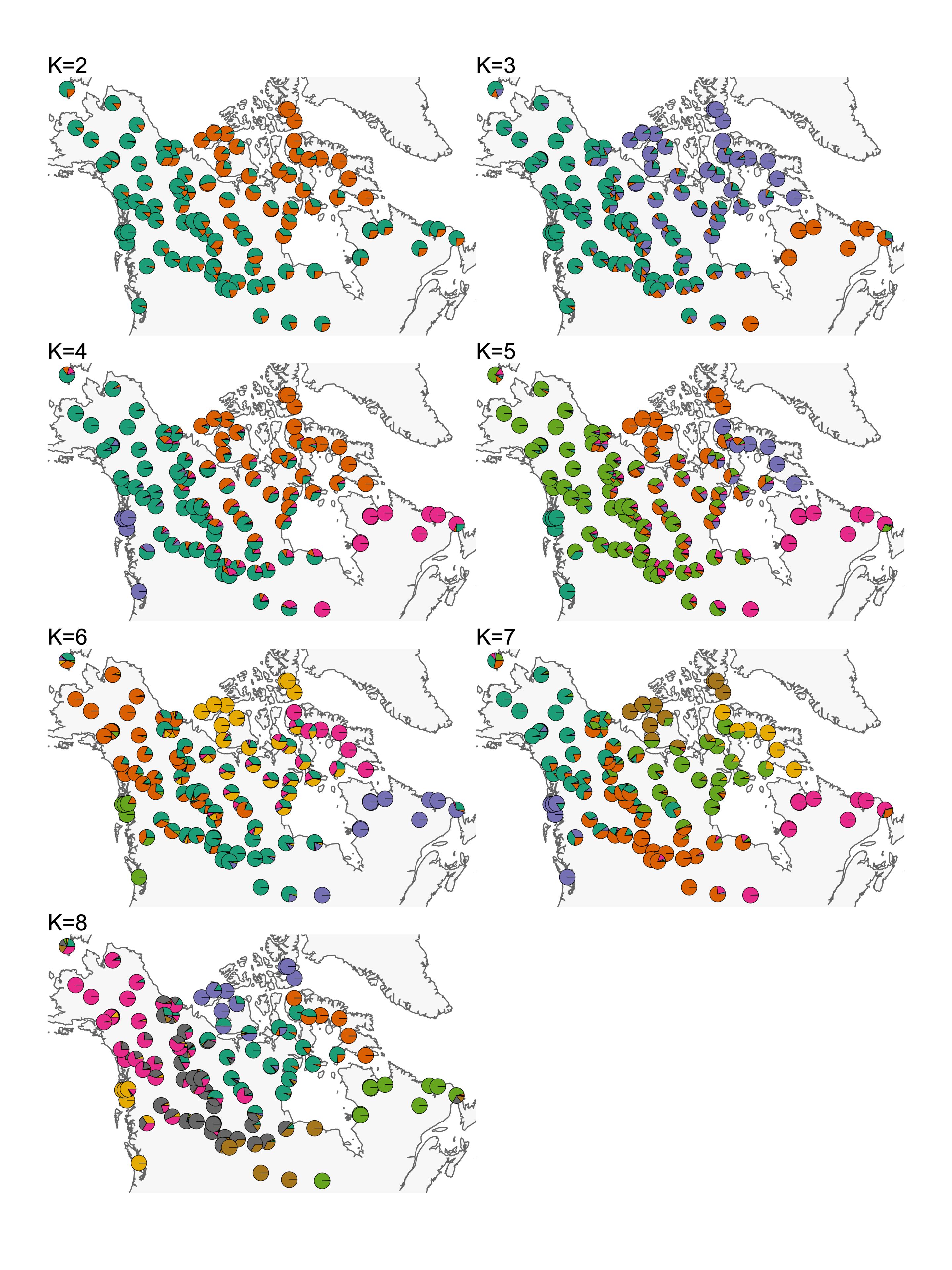

Supplement: S6 Fig — The base map is drawn using shape files generated by Cartopy (with the base layer available at https://www.naturalearthdata.com/download/50m/physical/ne_50m_land.zip, [36]). (TIF) [file pgen.1011612.s008.tif]

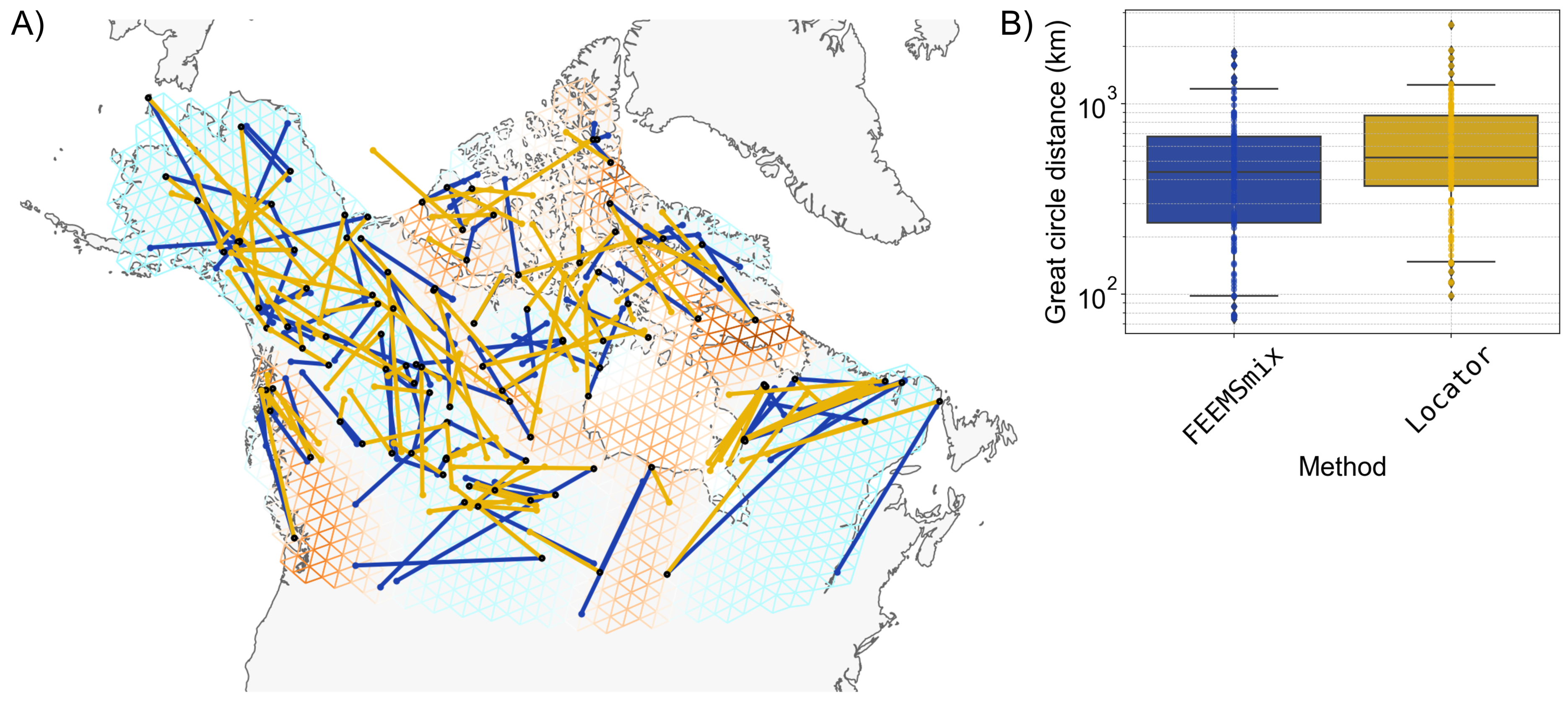

Supplement: S7 Fig — We see comparable results between the two methods, with slightly better performance in FEEMSmix (decrease in median error of approx. 100 km, though high error with both methods indicate how mobile wolves tend to be). True sample locations are shown as black points and the predicted locations are shown in the color corresponding to each method. An interesting point to note here is that the predicted locations from either method never cross the migration barriers as estimated by FEEMS, also indicating a correlation in predicted location (average cosine similarity of ∼0.5). The base map is drawn using shape files generated by Cartopy (with the base layer available at https://www.naturalearthdata.com/download/50m/physical/ne_50m_land.zip, [36]). (TIF) [file pgen.1011612.s009.tif]

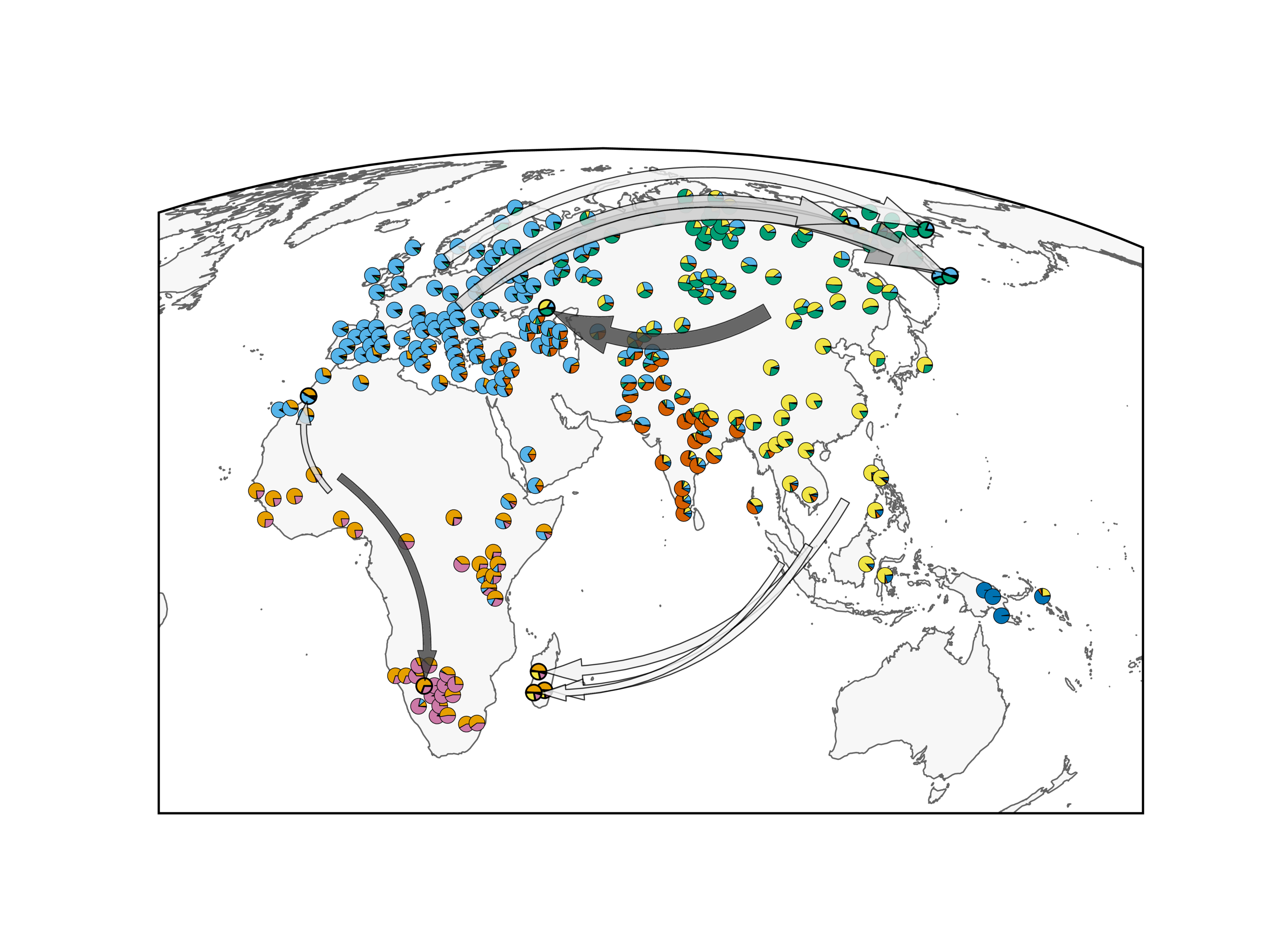

Supplement: S8 Fig — The base map is drawn using shape files generated by Cartopy (with the base layer available at https://www.naturalearthdata.com/download/50m/physical/ne_50m_land.zip, [36]). (TIF) [file pgen.1011612.s010.tif]

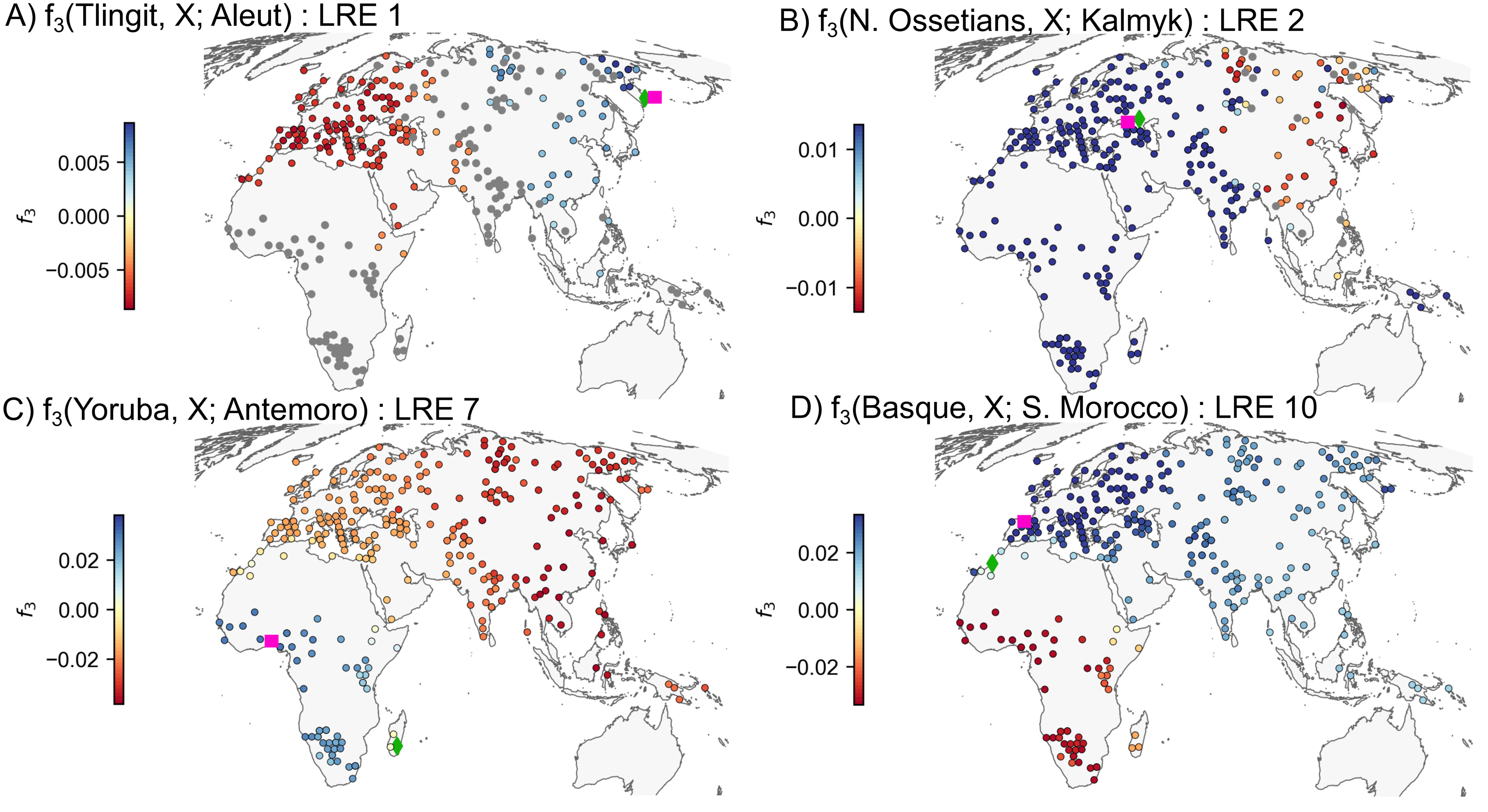

Supplement: S9 Fig — Here, A is the reference population, X is a testing population and T is the target population (i.e., destination deme). We observe that populations with the most negative f3 values (dark red) in each case are found in similar locations to the paired source demes inferred by FEEMSmix (in Fig 5B and 5C in the main text). The location of T is displayed as a green diamond and the location of the corresponding A is shown as a pink square. The base map is drawn using shape files generated by Cartopy (with the base layer available at https://www.naturalearthdata.com/download/50m/physical/ne_50m_land.zip, [36]). (TIF) [file pgen.1011612.s011.tif]

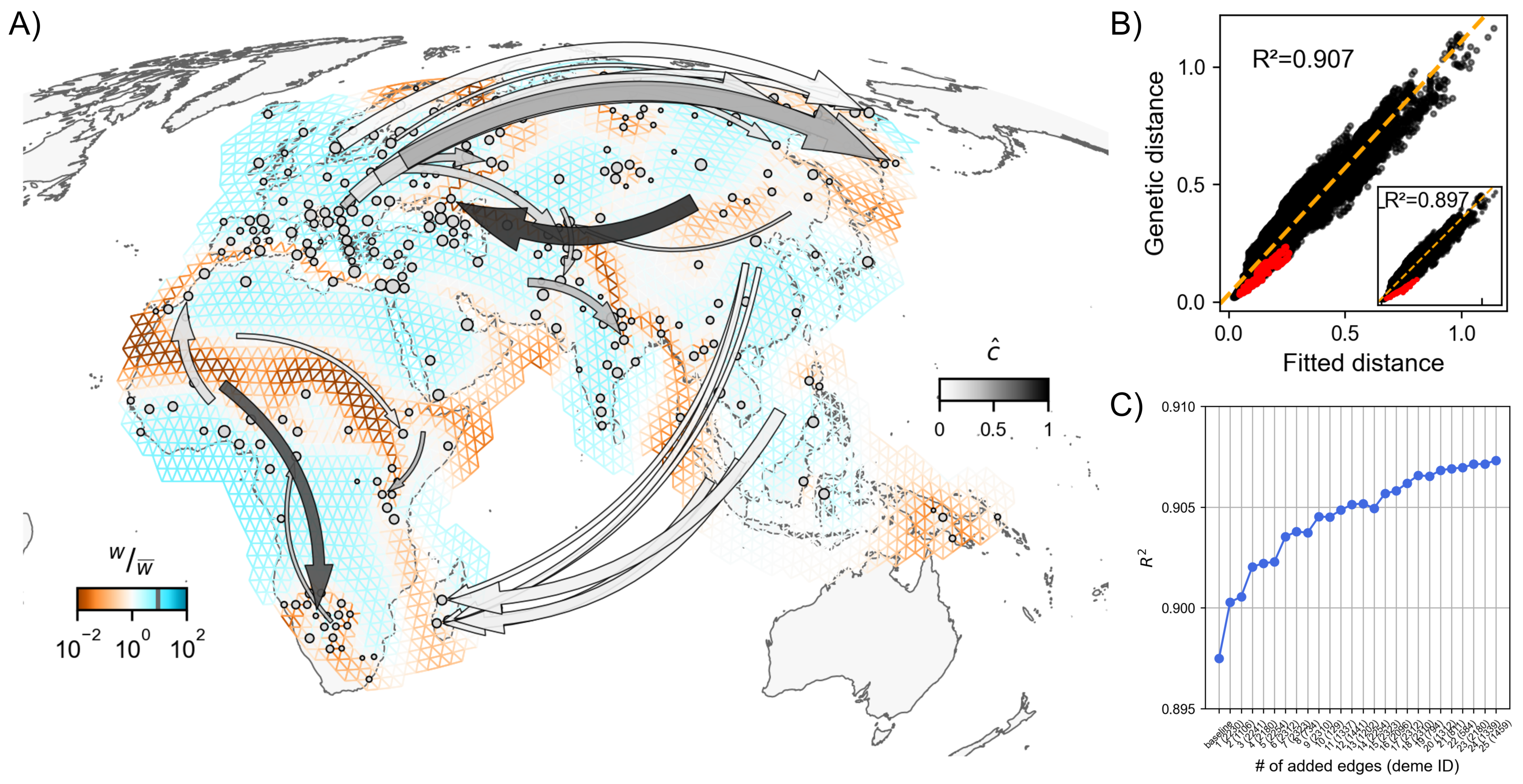

Supplement: S10 Fig — The base map is drawn using shape files generated by Cartopy (with the base layer available at https://www.naturalearthdata.com/download/50m/physical/ne_50m_land.zip, [36]). (TIF) [file pgen.1011612.s012.tif]

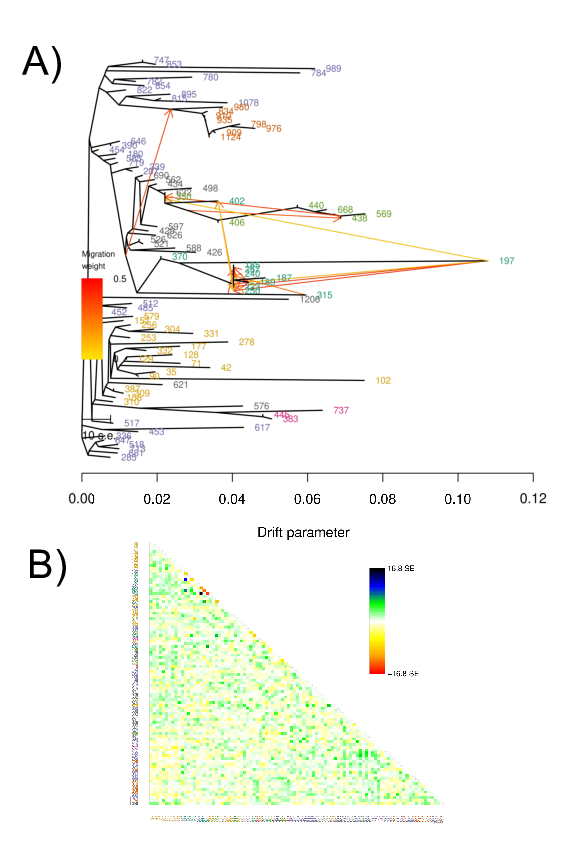

Supplement: S11 Fig — (TIF) [file pgen.1011612.s013.tif]

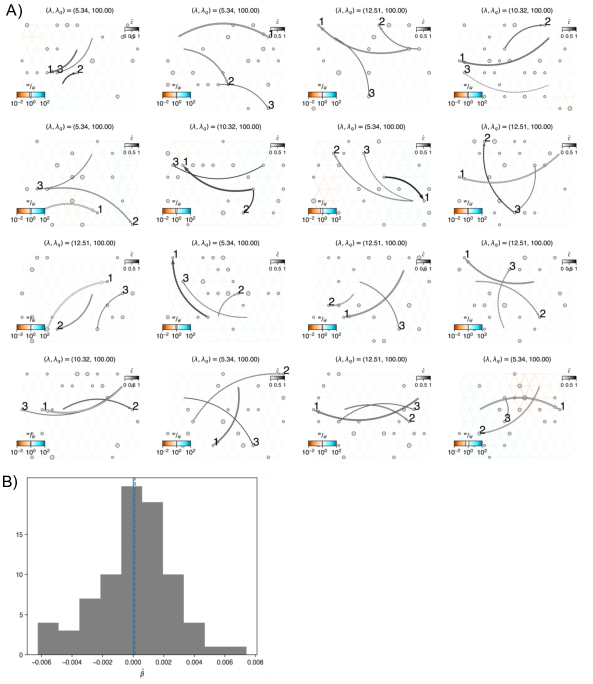

Supplement: S12 Fig — In A), we observe that FEEMS estimates a flat migration surface across all replicates, as expected. Out of 30 simulation replicates, the top LRE is found to show Lr>10 in 35%,C95=[22%,50%] of all simulations, and we note that fitting the model to data with panmixia rather than isolation-by-distance makes evaluation of this outcome difficult (in some sense migration is all long-range in a panmictic population). In B), we show that β^≈0 [−0.0006,0.0006] across all simulation replicates, reinforcing that there is no geographic structure in the genetic data. (TIF) [file pgen.1011612.s014.tif]

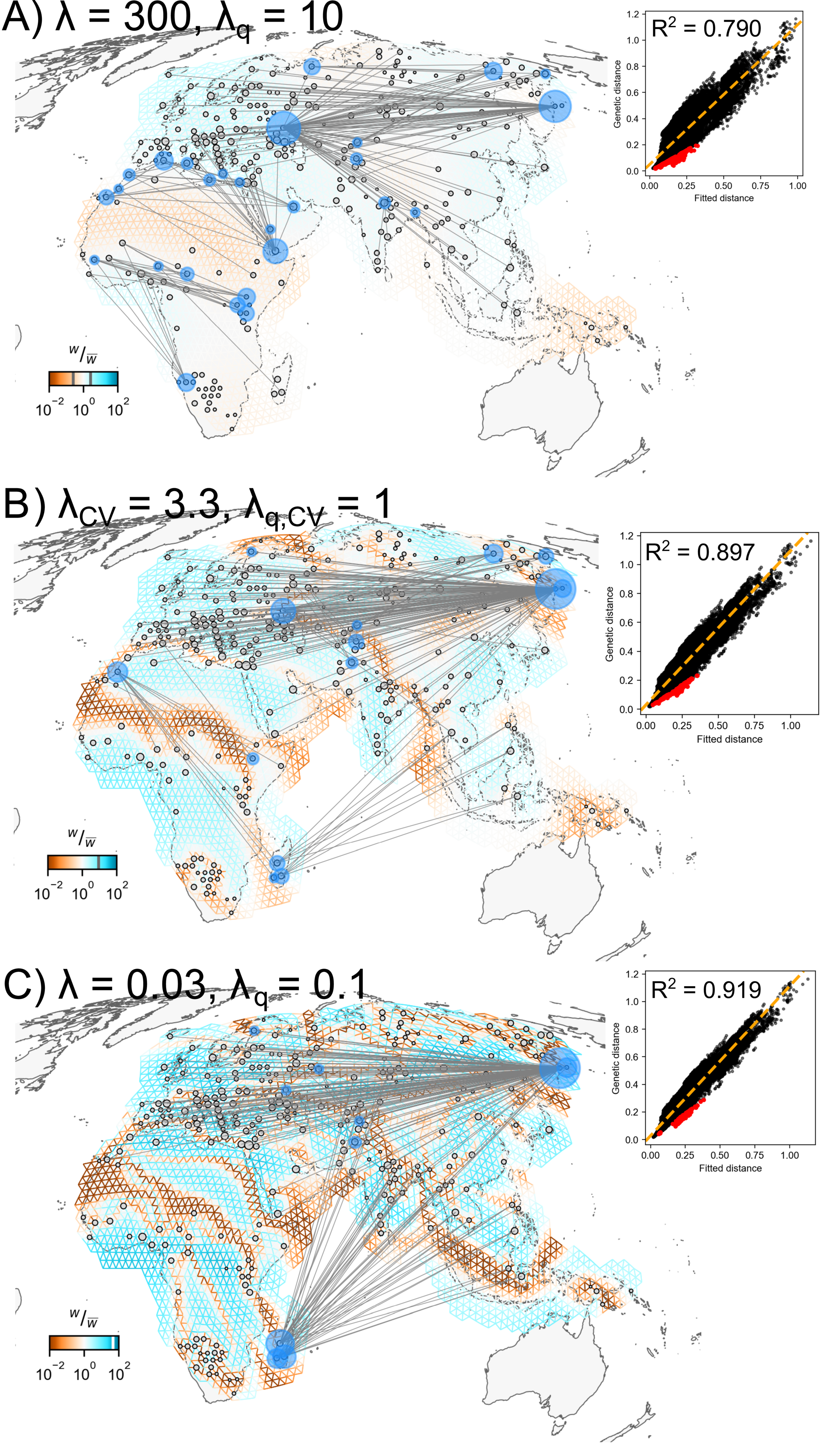

Supplement: S13 Fig — With decreasing λ values, we see new demes being implicated as outliers in the fit, that disappear with a very low λ value. This is likely due to overfitting with low λ, i.e., the baseline FEEMS fit connects two distant but similar demes via a snaking migration corridor connecting them, hence modeling away any residual. For instance, the Kalmyk individuals are no longer found as outliers, as they are connected to individuals further east through a migration corridor that punctuates a region of low inferred migration that is inferred with higher λ values. As expected, the baseline model R2 increases with decreasing λ, which is why leave-one-out cross validation is used to choose the optimal value for the λ’s. The base map is drawn using shape files generated by Cartopy (with the base layer available at https://www.naturalearthdata.com/download/50m/physical/ne_50m_land.zip, [36]). (TIF) [file pgen.1011612.s015.tif]

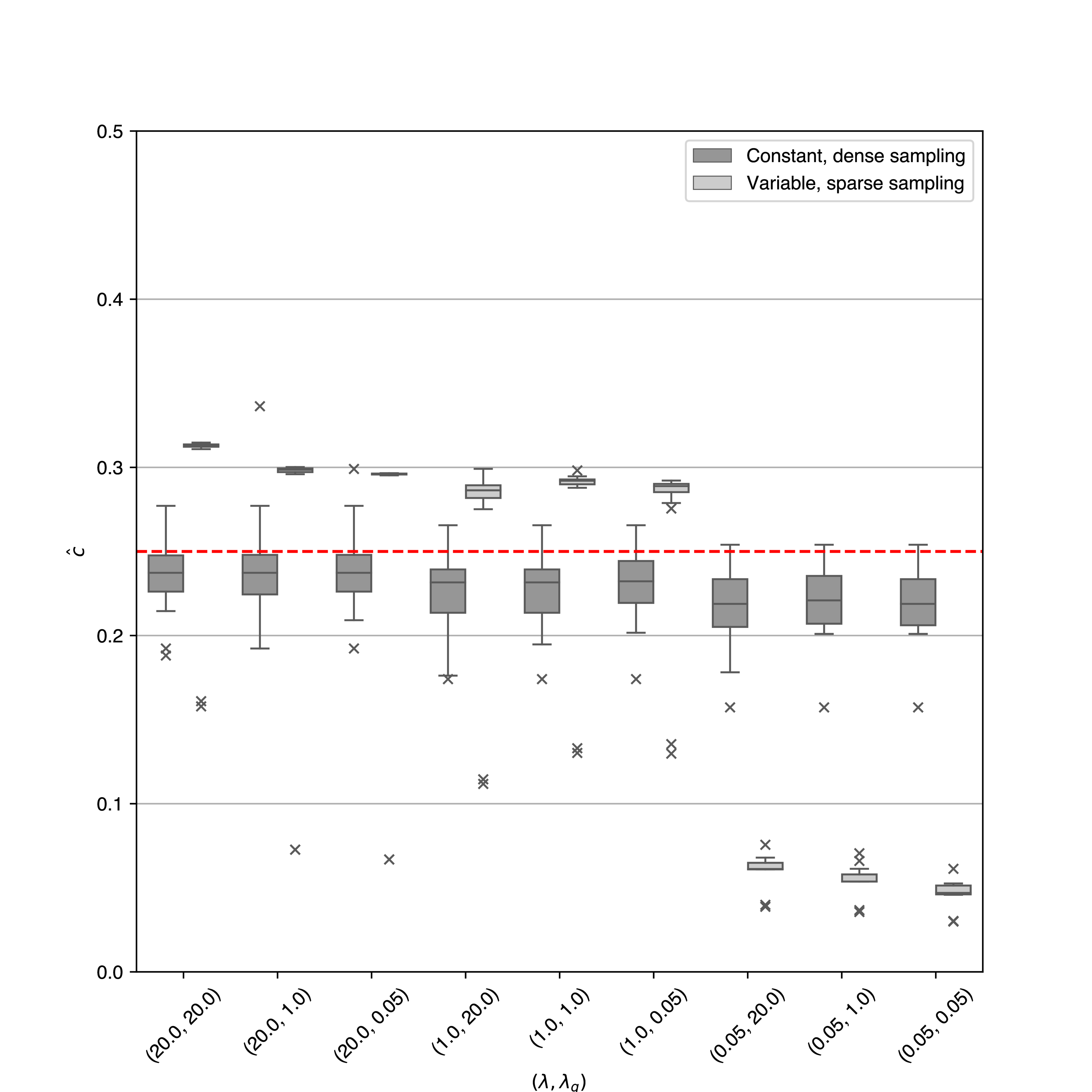

Supplement: S14 Fig — The true simulated strength is marked as a red line at 0.25. We observe that there is little to no difference in estimated values across different tuning parameter combinations in the case of constant, dense sampling, but quite a big difference in the case of variable, sparse sampling. Essentially, for very small values of λ, FEEMS overfits the patterns in the data (see the more disjoint maps in S4 Fig for a visualization of this), leading to very little extra residual, and in turn, leading to an underestimation of the strength of long-range gene flow. The most common pair of tuning parameter values chosen by the cross-validation procedure was (20.0,100.0) for the dense scenario (similar to (20.0,20.0) in the figure) and (0.3,1.0) for the sparse scenario (similar to (1.0,1.0) in the figure). (TIF) [file pgen.1011612.s016.tif]

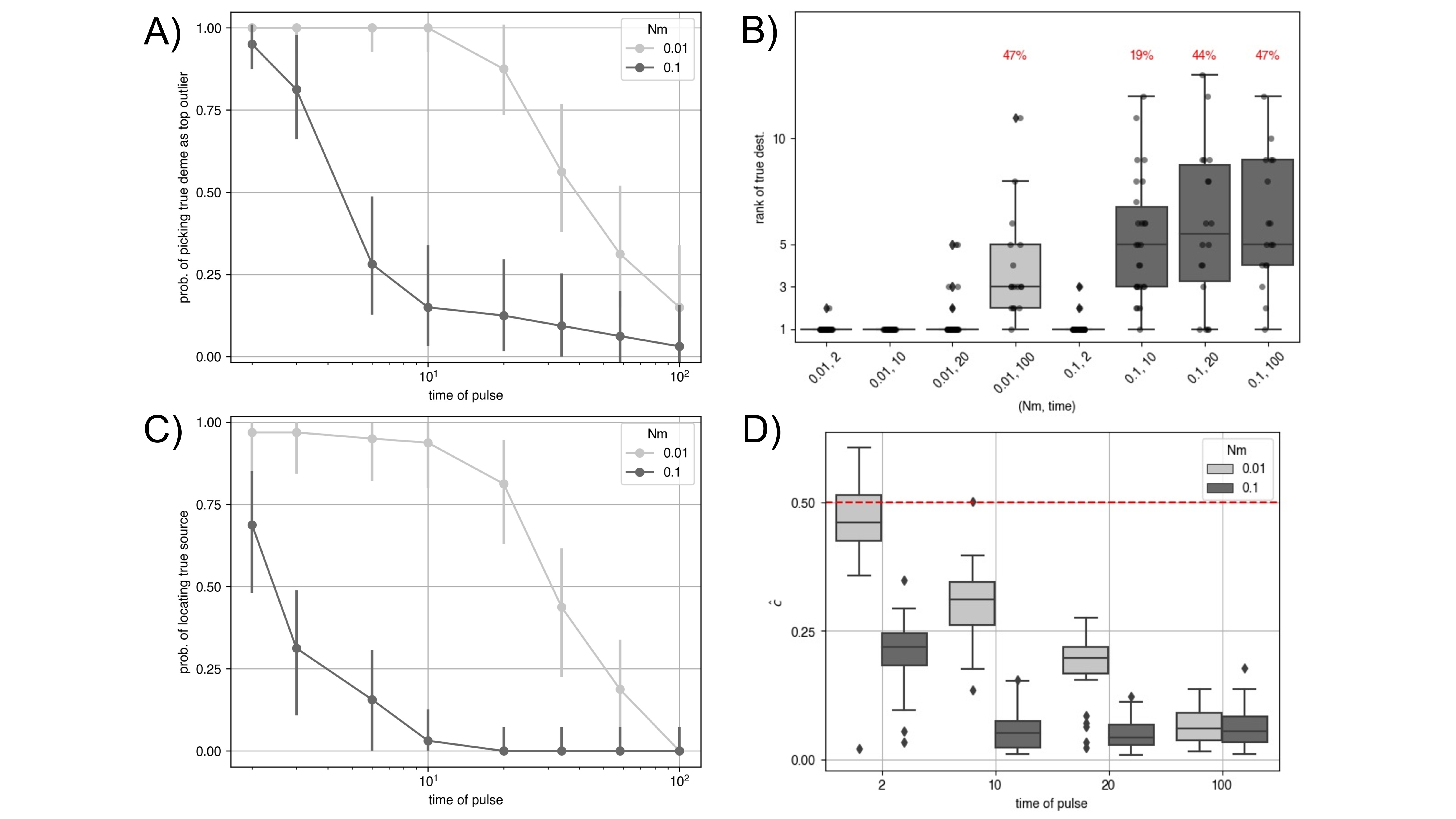

Supplement: S16 Fig — Overall, with increasing background migration and older admixture pulse, there is a washing out of long-range gene flow signal (as expected, see also S15 Fig). In A), we see that the model’s ability to pick the true destination deme as the top outlier drops off after a certain time back in the past. In B), we see that in certain regimes, the true deme is not implicated in the top 10 largest outliers as found by FEEMSmix, indicating a loss of the signal. In C), we see that with weak background migration, the model can pick out the true source of the long-range gene flow in about ∼80% of the replicates when the true destination is implicated. However, this performance drops off sharply under a higher level of background migration. Finally, in D), we see the characteristic decay in the estimates for the source fraction for older admixture pulses and stronger background gene-flow due to the diffusion of lineages across the surface (similar to S15 Fig). (TIF) [file pgen.1011612.s018.tif]

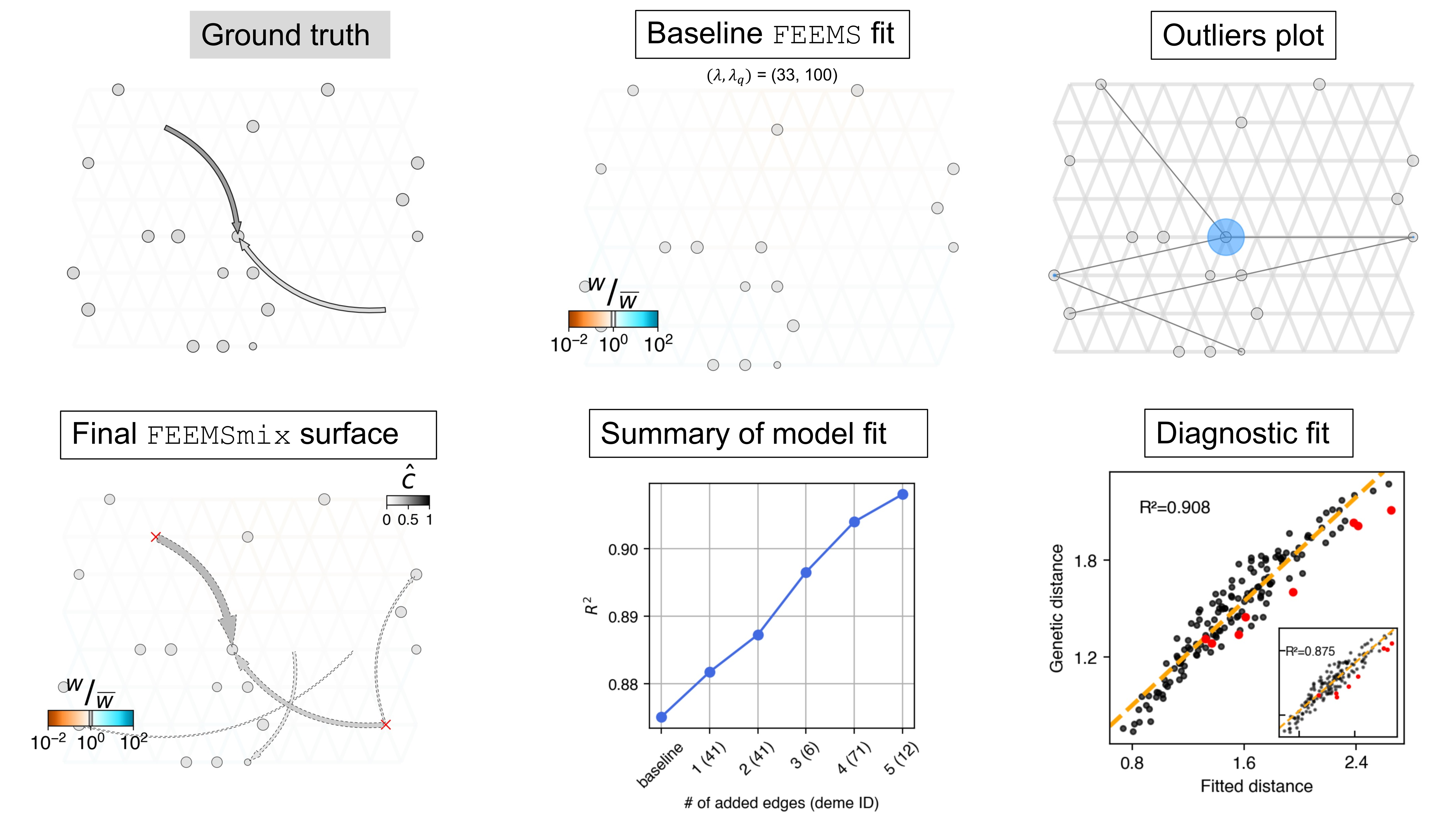

Supplement: S17 Fig — In Ground truth, we show that there are two simulated long-range gene flow events with differing strengths (NW source: c1 = 0.5, SE source: c2 = 0.25) from diagonally opposite parts of the habitat. We see the two regions with the true sources (in red crosses) is found exactly by FEEMSmix across the first two LREs with low error (c^1≈0.45,c^2≈0.3) on adding K = 5 edges. Though, we also observe that in this setting, the Lr statistic does not show large values as one might for two true LREs. (TIF) [file pgen.1011612.s019.tif]

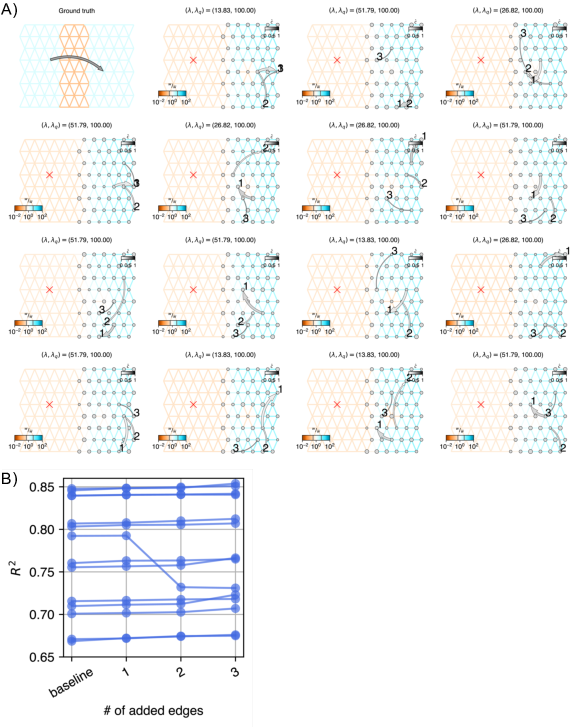

Supplement: S19 Fig — This presents an extremely challenging case. In A), we observe that the simulated long-range event is never captured, though the existence of a ‘pinwheel’-like pattern around the destination deme indicates an interesting signal. Additionally, FEEMS also fails to capture the migration corridor on the west side of the habitat. In B), we observe that adding any number of edges barely increases the fit to the data. Without sampling the source, there is apparently little gain to add an LRE in terms of R2. All added LREs pass the Lr > 10 threshold as there is still systematic structure that is not captured on the habitat. (TIF) [file pgen.1011612.s021.tif]

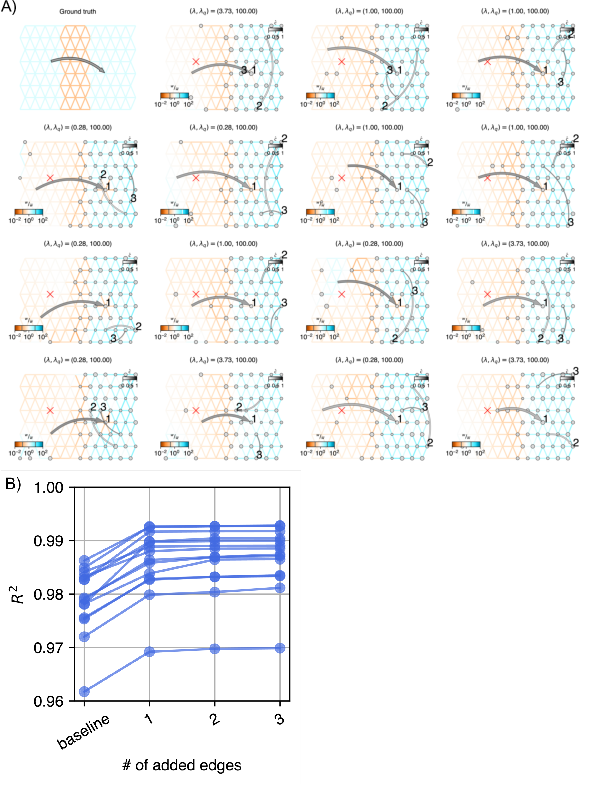

Supplement: S20 Fig — This also presents a very challenging case, but slightly less than in S19A Fig. In A), we observe that the simulated long-range event is always captured as the top event, indicating that in this setting even a small amount of sampling is sufficient to detect the long-range event. However, FEEMS still fails to capture the migration corridor on the west side of the habitat. In B), we observe that adding the first LRE provides a significant improvement in fitting the data with a plateau observed after K = 1 (similar to S4B Fig and improved compared to S19B Fig). With regard to the LREs, here too, we observe a similar behavior as in S19 Fig wherein all edges show Lr > 10. (TIF) [file pgen.1011612.s022.tif]

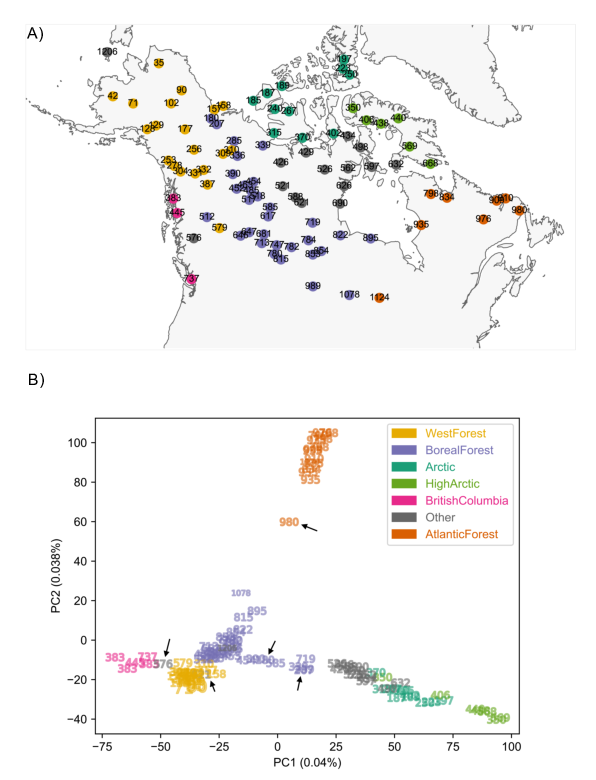

Supplement: S23 Fig — Deme 576 (also marked with a black arrow) might visually appear to be an outlier here, but its placement in PC space is in accordance with its geographic position. (TIF) [file pgen.1011612.s025.tif]

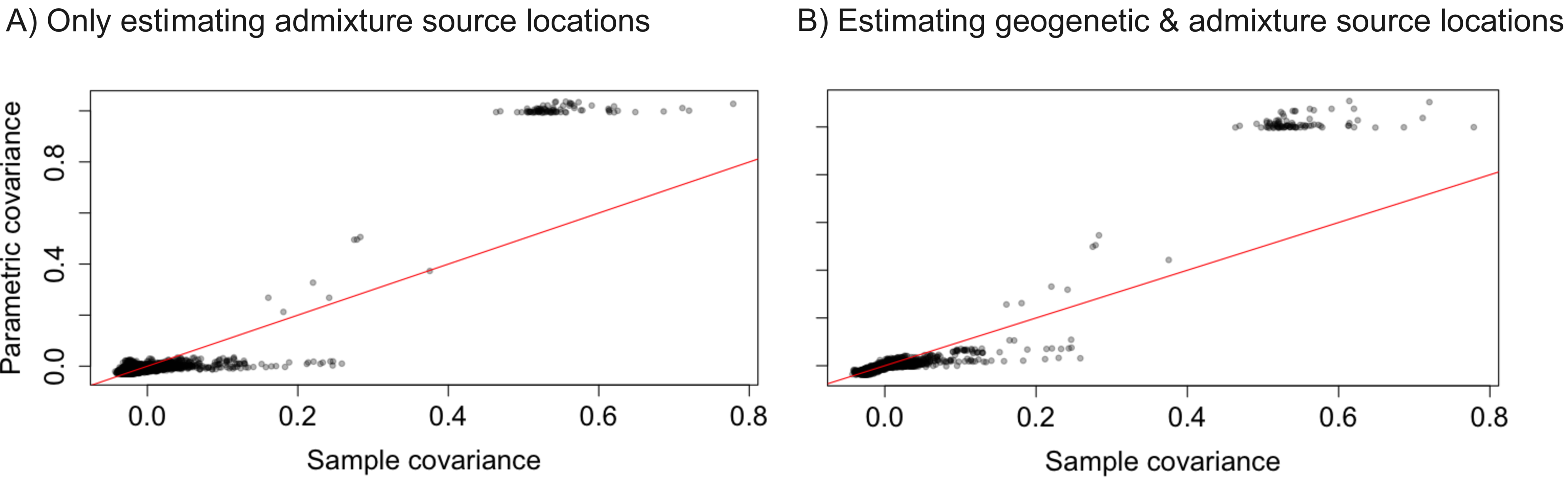

Supplement: S24 Fig — Both modes produce R2>0.9, and show a somewhat step-like pattern in the fits. (TIF) [file pgen.1011612.s026.tif]

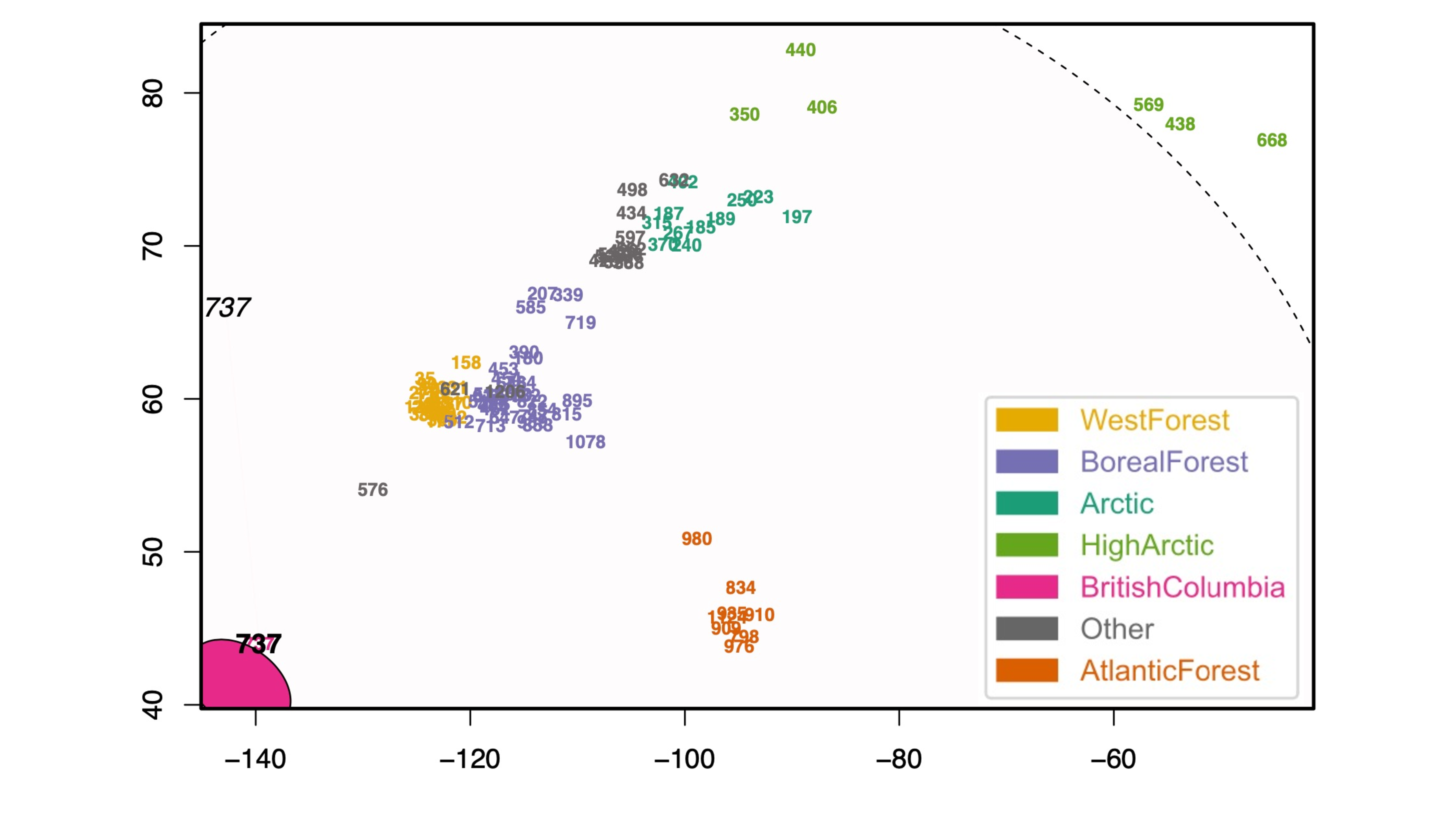

Supplement: S25 Fig — We see that the two spatial axes of location places these demes in a manner similar to PC1 & PC2 in S23B Fig. The location of the sampled demes correlates with their ‘Ecotypic’ classification (in S23A Fig). Only deme 737 is implicated in an admixture event, with the location of the source extending over the entire habitat. In all cases, we see that the 95% credible intervals for the source locations span the entire ‘geo-genetic’ space with the maximum a posteriori estimates differing from the results from running a different mode in S26 Fig. (TIF) [file pgen.1011612.s027.tif]

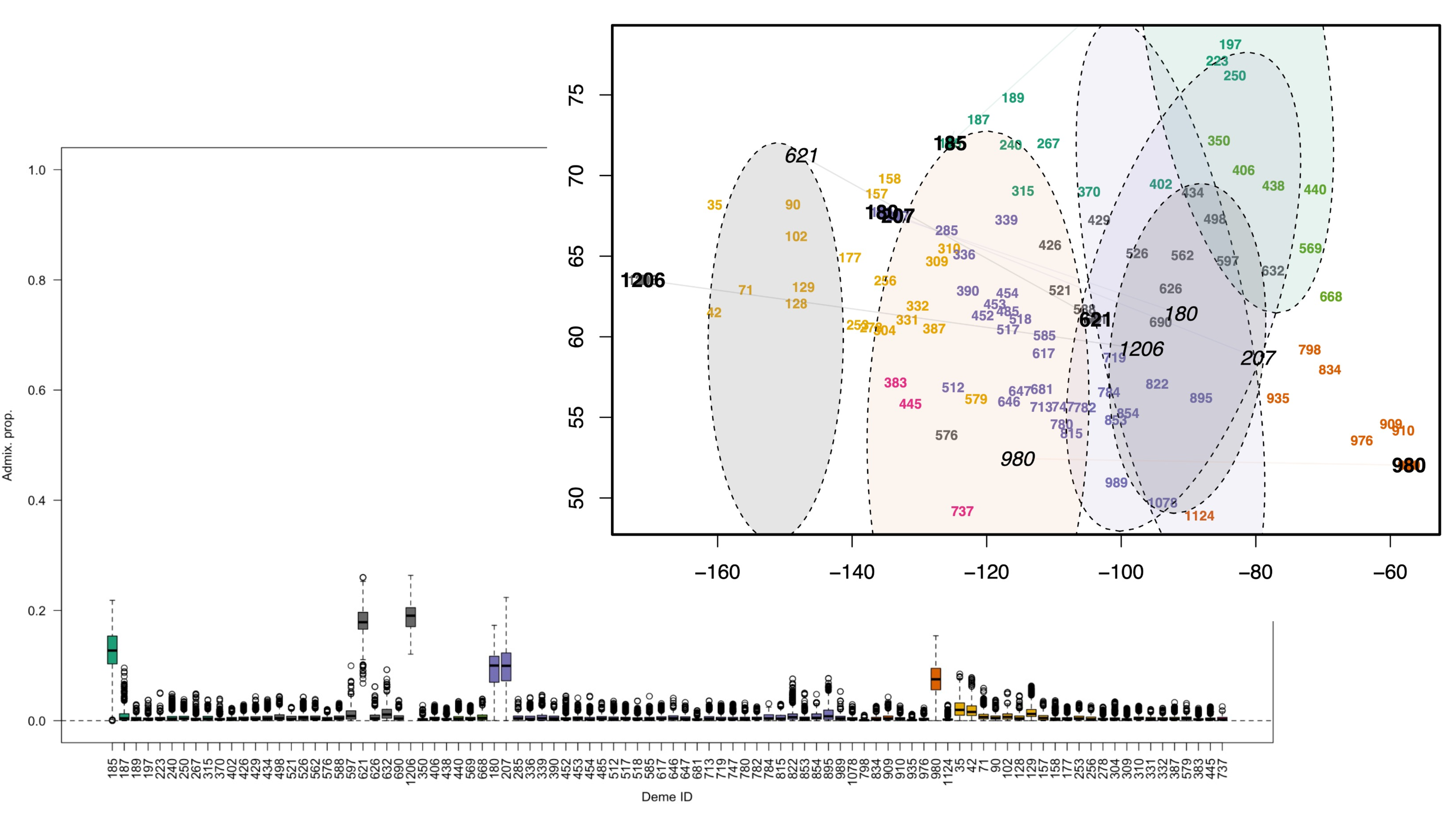

Supplement: S26 Fig — In the inset plot, we see similar results with FEEMSmix, both in terms of the identity of the putative destination demes and the location of the sources of these admixture events. This similarity is also consistent with the results from ADMIXTURE. We also display the posterior distributions of the admixture proportions (as boxplots) of the events shown in ellipses in the inset plot. (TIF) [file pgen.1011612.s028.tif]

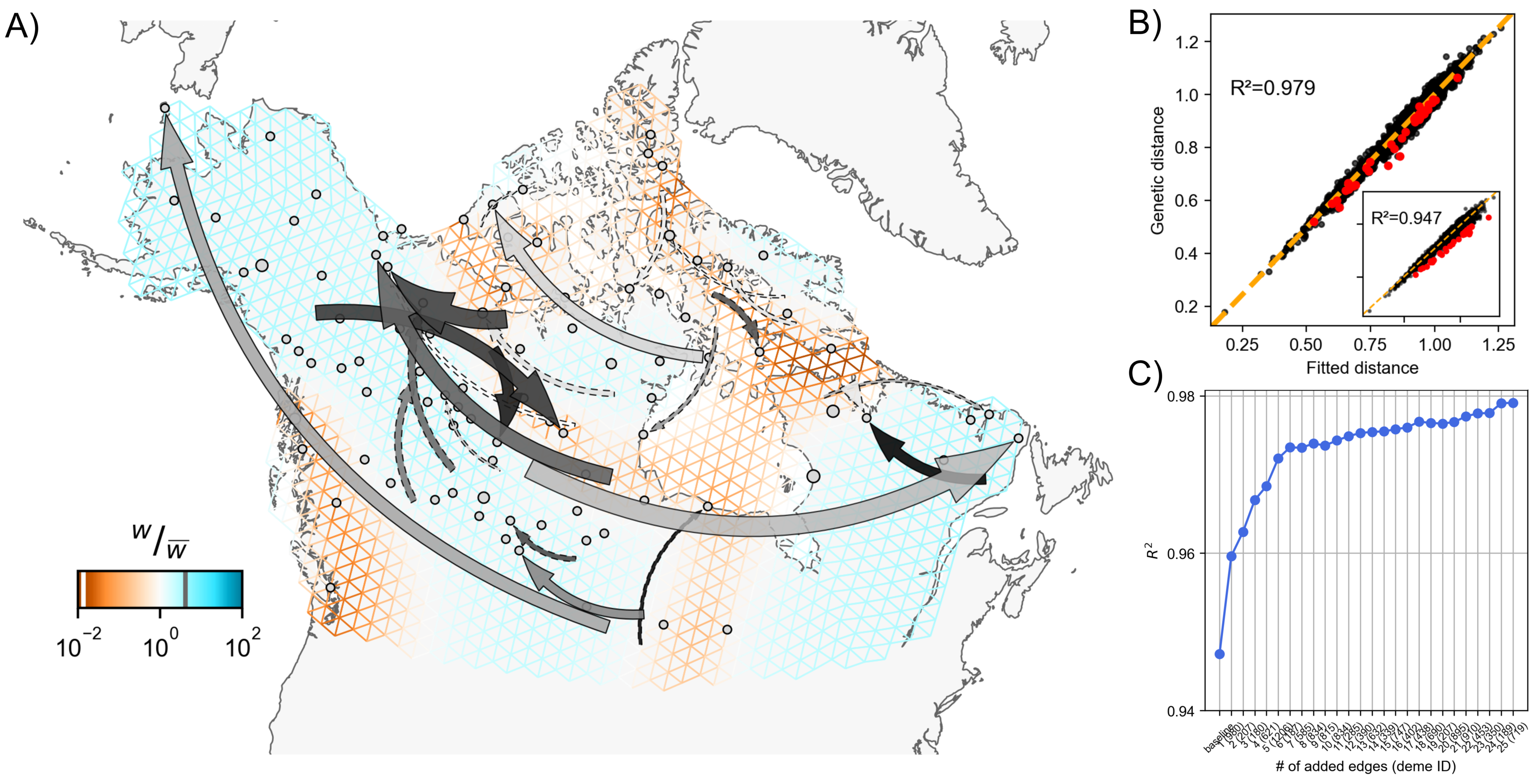

Supplement: S27 Fig — The base map is drawn using shape files generated by Cartopy (with the base layer available at https://www.naturalearthdata.com/download/50m/physical/ne_50m_land.zip, [36]). (TIF) [file pgen.1011612.s029.tif]

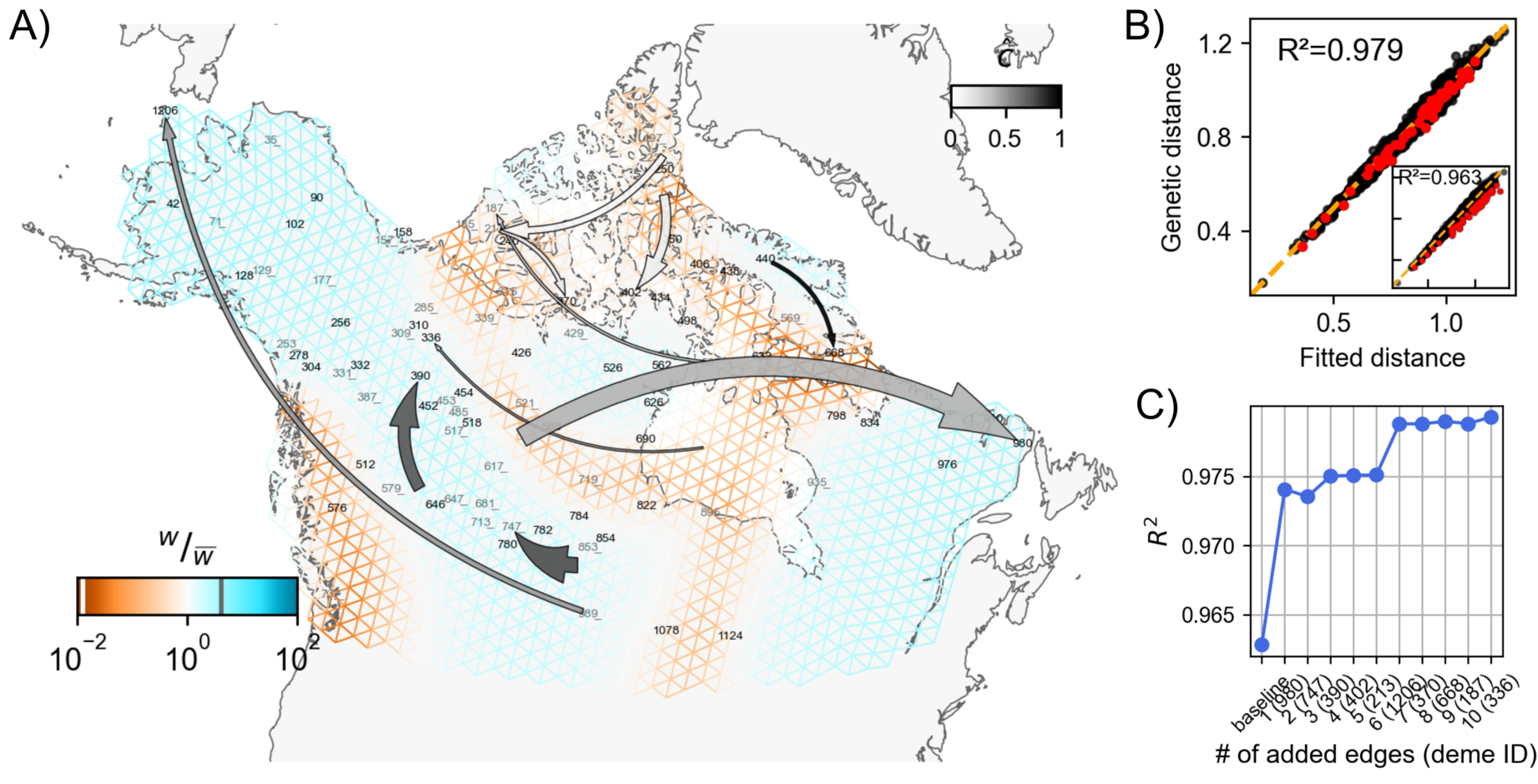

Supplement: S28 Fig — The base map is drawn using shape files generated by Cartopy (with the base layer available at https://www.naturalearthdata.com/download/50m/physical/ne_50m_land.zip, [36]). (TIF) [file pgen.1011612.s030.tif]
