# Supplementary material for: Jointly representing long-range genetic similarity and spatially heterogeneous isolation-by-distance
Source: PLoS Genet. 2025 Sep 16;21(9):e1011612. doi: 10.1371/journal.pgen.1011612 (PMC12453258; doi:10.1371/journal.pgen.1011612)
Supplement: Attachment — Submitted filename: response_to_reviewers.pdf [file pgen.1011612.s031.pdf]

Responses to reviewer comments

We would like to thank the reviewers, and the editor, for their detailed reading of our manuscript, and for their comments. The manuscript has been substantially improved through these revisions, and we appreciate the opportunity to resubmit.

We have responded to all reviewers' concerns with the most substantive changes being the addition of a new methodology for assessing support for long-range edges and extensive new simulations to examine the robustness of the method.

Below, we provide detailed responses to each of the reviewers' comments. Reviewer comments are shown in black and our responses are shown in blue. To ease review of changes to our manuscript, we have included a PDF of the manuscript with the additions highlighted in blue and the deletions marked in red and placed in the footnotes.

I have obtained 3 expert reviews, which are included below. I agree with the reviewers that the proposed method is a nice extension to existing work and that it would be a useful tool in the empiricist's spatial population genetic toolkit. Although I ask you to respond to each reviewer comment, a few points deserve special attention as you revise.

First, I think more could be done to elevate this work and increase its impact by making it of interest to a broader audience. This is a point that came up in several reviews, and I think there are a number of ways to do it, including broadening the scope of the Introduction or elaborating on the method to incorporate a temporal component to the long-distance edges.

We appreciate the encouragement to improve the manuscript to elevate the work and enhance its impact. Following the suggestion to broaden the scope of the Introduction and reviewer 2's comments, we have updated the Introduction to provide more background on the existing methods in a way that makes the paper more accessible. Regarding an elaboration of a temporal component to the long-distance edges, we still believe this is better saved for future work (we attempted to do it prior to our original submission, but it is quite challenging to manage the computations involved). That said, in the revision, partly also in response to reviewer 3's call for more simulations, we provide results from new simulations where the age of the pulse event is varied to guide users in understanding the performance of the framework in those settings.

Another point that all reviewers brought up is that more exploration of the statistical behavior of the addition of LREs is warranted. How should a user know when to stop adding edges? What should we make of the jumps in likelihood that come from adding certain LREs. And, an additional point - what about LRE "false positives?"

We appreciate this is a key area of interest and challenge. For background, we note how `TreeMix` took a similar approach to our initial submission in having a user-specified K number of edges to explore. `SpaceMix` can fit several alternative models including a "model 4" with an admixture component to all sampled populations, and has users choose between models.

For the revision, we explored the development of an automated stopping criterion for adding edges based on the likelihood ratio between a model where the residuals are uni-modal vs bi-modal. The models are not strictly nested and because we are using penalized

likelihoods for the model fitting, it is difficult to use any standard model selection criteria rigorously. We explored using the parametric bootstrap but it is too computationally costly. As such, we settled on the use of a likelihood ratio test statistic (which we denoted L_r, see below) as the basis of a heuristic stopping criterion (We focus on this likelihood ratio statistic as it would also underlie ΔAIC/BIC model selection criteria and we are only choosing between two models). Perhaps not surprisingly for a difficult model selection problem, we found this heuristic approach to perform with variable success. As such we continue to encourage user-guided approach exploring different choices for K, as in `TreeMix`, but we feel the revision is a substantial improvement in that we now fit models for the residuals and report a likelihood ratio statistic that aids in interpretation.

In more detail, we assess long-range edge support sequentially by comparing two competing models for the distribution of the deviation statistic (see Fig 1):

- **Model 1: Null**: the deviation follows a univariate Normal distribution (no systematic structure remains)
- **Model 2: Bimodal residuals**: the deviation follows a mixture of two univariate Normal distributions (systematic structure present, suggesting long-range connectivity)

Support for each model is evaluated using the statistic $L_r = -2\ln(\hat{L}_2/\hat{L}_1)$, and we use a threshold of > 10 as to identify whether adding an edge has strong support. This roughly corresponds to $p \approx 0.01$ under a chi-squared distribution with three degrees-of-freedom, if asymptotic theory held, which it does not here, so we downplay this interpretation in the text.We explored using our stopping criterion approach to add LREs until the deviation can be modeled as a single univariate Normal distribution. We found that a stopping criterion based on adding edges while $L_r > 10$ performs best in the simplest simulations with just a single long-range gene flow event (see Fig S3-S4). However, in more complex scenarios with multiple long-range gene flow events, we find the approach to be too conservative and can fail to capture even a single event (see Fig S21-S22).

So while this approach improves our metrics of quantitative evidence for detecting outliers in the data, though we acknowledge the inherent challenges in model selection and disentangling overlapping signals in spatially structured populations. Based on the results across a range of simulations, we still favor the `TreeMix` (and our previous) approach, but the manuscript and software still provide summaries from the stopping criterion approach as it is informative. Overall, we feel this provides a more rigorous statistical characterization of the edges and improves the manuscript and the `FEEMSmix` software as well.

Reviewer #1

This is an interesting and well-presented manuscript describing an extension to the EEMS/FEEMS method called FEEMSmix. FEEMSmix models long-range genetic similarity among samples by identifying outlier residuals from a FEEMS fit and adding additional edges to the FEEMS topology along which long-range gene flow can occur. FEEMSmix fills a definite need for

describing spatial patterns in genetic variation data, and I think it will be a welcome addition to the spatial population genetics toolkit.

Thank you for your kind words!

I think the big concern with this type approach is identifying which of the long range edges (LRE) inferred by FEEMSmix represent historical long-range gene flow events and which represent overfitting by the model. In many cases the amount of variance explained by a FEEMS model is quite high (90 percent or more for both wolves and humans), and in those circumstances it seems like there is a danger of FEEMSmix fitting parameters (=LRE) to random noise in the data. The authors provide reasonable, corroborated explanations for some of the LRE identified in their case studies. But presumably there are many LRE for which there are no obvious explanations, and FEEMSmix has the capability to keep adding LRE until the model fits the data perfectly. What do we do with all these LRE? How do we tell which LRE warrant inferences of long-range migration and which do not? Are there formal model comparison tests that are applicable and that could be used to compare FEEMSmix to FEEMS to help decide if the additional complexity added by FEEMSmix is justified on information theoretic grounds? I don't think this issue detracts from the general utility of the model, but I think more discussion and guidance from the authors would be useful for potential users of the method, perhaps when they are discussing additional caveats of FEEMSmix in the discussion.

Well stated. This is a challenge faced also by `TreeMix` and `SpaceMix` and one that we grappled with as we developed the method and prepared the manuscript. For the revision, we have made a new effort to address the problem (see response to editor above). While not a panacea – particularly in complex multi-event scenarios – this framework is an improvement with regards to understanding the statistical support for LREs and can provide an indicator to users to stop adding edges.

Minor comments:

Line 233, what does "100% [93%, 100%]" mean? This style of reporting appears throughout this section and I found it confusing. Is it the most frequent value with the range of values appearing in brackets? Is it an estimated mean and confidence interval?

This was unclear, we agree – This bracket notation represents the 95% confidence intervals calculated using the Clopper-Pearson method given the number of trials (i.e. simulation replicates). We apologize for the confusion as this wasn't mentioned in the text. We now include a sentence (lines 451-452) to explain this in the main text.

Line 241, how well is the true source identified when the destination deme is *not* fixed to the true one? It seems like a better performance metric would be setting the destination deme to whatever FEEMSmix thinks is the most likely.

Excellent point. We have modified Fig 2 and reworded the Results to reflect the performance in our simulations under the requested case. As expected, the performance is somewhat worse when detecting the location of the source as seen in Fig 2A & C, but is comparable to the case when we use the true destination deme with strong gene flow. This highlights an expected regime in which the model performs quite poorly: very weak gene flow ($c = 0.05$) with sparse sampling. Though the mean source location is misestimated in

this case, the coverage statistic in Fig 2B highlights that the confidence intervals are wide enough to capture the true source.

Eq 1: can you elaborate here? You have written T'[sd] = cT[sd] + (1-c)T[ss] but my brain wants it to be T'[sd] = (1-c)T[sd] + cT[ss]. What am I missing? Also, these apply only to sampled demes, is that correct? Or can s be unsampled?

Thanks for catching that typo. Actually, Eq (1) captures the dynamics even in the case the source is unsampled. Practically speaking, the only thing that changes is how T_{ss} is estimated in this case: we use a kriging-based approach compared to using the penalized likelihood estimates directly from `FEEMS` (see *Approximation of pairwise coalescent times in an unsampled deme*).

It would also be helpful to unpack the post-event expected genetic distance matrix equation a little, although this could be in the supplement.

Great idea: we now include a short paragraph following the equations to unpack the model a little bit more (lines 331-345). We have also condensed the different cases into just two to make the equation more readable now. Generally speaking, the elements of this matrix don't have the same, easy intuition as seen with the coalescent times, so this makes readability slightly challenging.

Line 532, what is the separate equation?

We apologize for not being clear. There is no longer a separate equation anymore as it can be absorbed into one of the two cases above.

line 541, does it scale the units to expected distances or doesn't it? The "should" qualifier is odd here.

It does scale the units, so we replaced *should* in the main text with *will*.

Regarding future directions, could one imagine a version of FEEMSmix where edges are removed from the baseline FEEMS fit? For example, imagine a set of populations separated by unsuitable habitat such that all gene flow between them occurs by occasional long-range dispersal events across an unsuitable matrix. In that case, the FEEMS assumption of migration through locally interacting demes spanning the unsuitable habitat is incorrect, and a LRE (or set of LRE) inferred by FEEMSmix could replace the local edges in the baseline FEEMS fit. This is just speculation on my part, I'm not familiar enough with FEEMS to know if this is feasible.

This is a very interesting idea — we have not taken on implementing and testing such an idea here, but we now include this as a potential avenue for future work in the Discussion section (lines 738-745).

Reviewer #2

I'm a fan of the EEMS framework. It is used a lot by empirical researchers. Thus, it's nice to see it built on in productive ways. However, given the apparent similarity to spacemix it feels like the extension done here could have advanced things more substantially.

We appreciate the acknowledgment of the contribution. Our goal was largely to incorporate the advantages of `TreeMix` and `SpaceMix` into the `EEMS/FEEMS` framework. While we thought this would be a simple undertaking at first, it was a substantial project and we hope that may help with appreciating the work on its own terms.

The authors acknowledge: "This framework follows closely from existing methods that model the residual from an existing fit as a specialized admixture component (TreeMix, Pickrell & Pritchard (2012); MixMapper, Lipson et al. (2013); SpaceMix, Bradburd et al. (2016))." While treemix and mixmapper are conceptually similar, the spatial admixture framework seems to essentially be the spacemix framework. I'm excited to see these ideas implemented to EEMS, and there's lots of advantages to the EEMS framework (in terms of speed and interpretability). However, I think the authors should explain in a few sentences the admixture model that was implemented in Spacemix in their introduction, and where their spatial admixture model differs, as few readers will reread the earlier references.

This suggestion is important and well received. In the revised Introduction, we now reference the `SpaceMix` admixture model more explicitly and explain how our model is both similar and different with a couple of sentences (lines 95-107).

Given the similarity to previous methods, it is slight shame that the new method didn't go further in their model of instantaneous admixture. For example, if I'm understanding it correctly, only a single [node] on the grid receives the pulse of admixture, but the authors could've allowed some small number of adjacent [nodes] to share the source admixture (this is briefly mentioned in the discussion). One suggestion is that the authors could simulate under this model and show how this shows up in the model.

This point is well taken and we have added simulations of a few variants of this proposed scenario as part of the revision (Fig S21-S22).

For example, in the human data analysis there look to be quite a few arrows highlighting similar admixture routes, which could be due to this limitation of the model.

This is a valid question to raise regarding the human data analysis from Fig 5.To explore this, we simulated migration from one source to multiple neighboring demes under two potential cases: 1) the three destination demes are in an area of low effective migration (like LREs 1, 3, 4, 5), and 2) the three destination demes are in an area of high effective migration (like LREs 6, 7, 9). In both cases, we find it is possible for `FEEMSmix` to uniquely identify each event separately as a unique LRE, and more so in the case when each destination deme is within an area of low effective migration indicating high genetic distance between demes (see Fig S21-S22).

This mimics the results observed in the empirical human data, in which a separate LRE is fitted for each deme receiving admixture. However, without temporal information, we cannot distinguish whether these represent multiple independent admixture events or a single event followed by subsequent migration and drift. `FEEMSmix` models the unexpected genetic dissimilarity between pairs of demes and therefore cannot distinguish between multiple independent admixture events versus a single event followed by subsequent migration and drift——both scenarios would produce similar patterns of genetic covariance.

For the eastern Europe to western Russia/Siberia route, each of the four small populations are highly drifted in an area of low effective migration and it is plausible each long-range edge

captures recent admixture (following on previous work in Cardona et al 2014, Yunusbayev et al 2015, Mallick et al 2016). Whereas, for the south-eastern Asia to Madagascar route (Pierron et al 2014, 2017, Brucato et al 2016), this probably reflects a single event followed by subsequent drift or migration.

It's notable in the human data analysis that the improvement in R2 with number of admixture edges (Figure 5e) keeps almost flatlining and then making large jumps. Does this suggest that the procedure for choosing the next arrow to add is potentially sub-optimal?

This is an interesting hypothesis regarding the flatlining and large jumps seen in improvement. Doing an exhaustive search for the most optimal next LRE is prohibitive, but based on the reviewer's next comment we now explore an alternate procedure for choosing the next LRE.

If I am understanding correctly, the authors choose to prioritize fitting admixture arrows to demes that appear most often in their outlier pairs (line 175). Does this prioritize adding arrows to regions where there are many sample locations affected, which is good, but not take into account the magnitude of the outliers? I wonder if using a different metric to choose the arrows would help, e.g. that includes the number and magnitude of the outliers.

The suggestion for using both the number and magnitude of the residual of outliers is very useful and we have now incorporated this into the method. Previously, we were only using the number of outliers which was a discrete measure, but now we use a continuous aggregate statistic (lines 192-197) that sums over the magnitude of deviation and the number of times a deme has been implicated in choosing the next outlier. Though this only slightly improves our performance in choosing the next recipient deme, it does provide a conceptually more robust methodology (especially when deciding tie-breakers).

Reviewer #3

This manuscript presents an extension of the EEMS method that reconstructs spatial dispersal dynamics from the analysis of geo-referenced genetic data. More specifically, the new technique accommodates for long-range, recent migration events between demes besides "standard" IBD patterns. Simulations along with the analysis of two real data sets were performed in order to illustrate the relevance of the proposed approach.

Reconstructing past dispersal/migration events from the analysis of genetic data is a central endeavour in evolutionary biology and ecology. It is indeed paramount to our understanding of the forces shaping the spatial dynamics of related lineages during the course of their evolution. The EEMS framework provides graphical summaries that are straightforward to interpret, making that tool most useful to evolutionary biologists in practice. Relaxing the strict IBD assumption whereby long-range migrations are now authorised is a substantial improvement. Yet, assessing the relevance of the new approach requires further investigation in my opinion. I list below some points that may provide some guidance in that respect.

Thanks for your assessment of the work and suggestions for improvement.

Simulations

Current simulations do not provide enough evidence about the limitations of the proposed methods. In particular, FEEMSmix relies on the hypothesis of recent long-range migration of individuals from destination to source demes. The manuscript does not provide any specifics about the actual meaning of "recent" here. It is unfortunate as the power with which FEEMSmix detects long-range events probably depends on the age of those events.

To provide a tractable model, `FEEMSmix` models long-range gene flow as an instantaneous admixture pulse in the most recent generation. We now make this more clear in the description of the model (lines 291-293). In the presence of local gene flow after a long-range event, the signal of a more ancient long-range gene flow event will diffuse locally over the landscape with the rate of diffusion depending on the local migration rates. To make this more clear, we now conduct extensive simulations across a range of ages for the gene flow events. In Fig S15-S16, we show how there is a decay in this ability to detect the event with older admixture pulses and higher levels of local gene flow. This is also described in the Discussion section (lines 701-704)

Sampling considerations also probably matter. Indeed, data may no longer convey signal about the directionality of migration in situations where the recipient deme is isolated (e.g. ((A,B),(A,A,A,A)) suggests a recent migration from region A to B or a migration from B to A. Both scenarios are equally parsimonious, whereas ((B,B,A,B),(A,A,A,A)) is clearly indicative of a B to A migration).

Yes, in theory, with only two sampling locations, it would be hard to distinguish directionality under certain configurations of the data. However, with the typical sampling configurations that `FEEMS` is run with, we also have data points *around* the locations of long-range gene that help distinguish between the two directions. As evidence, in at least 10% of our sparse simulation replicates across *all* strengths of gene flow (in Fig 2, Fig S4A) there is only a single sampled individual at the destination and no sampled individuals at the source. But in *all* such cases, the method confidently estimates the direction of long-range gene flow to be in the same direction as the simulated.

On a related matter, dense and spare sampling are conducted in a uniform manner on the grid as far as I could understand. In practice, sampling is driven by practical considerations (accessibility, funding available, etc.), generally making it highly heterogeneous, i.e. non-uniform. Testing the robustness of the proposed technique to various (non-uniform) sampling patterns seems important if this method is to be applied to a broad variety of data sets.

This is a very useful practical consideration, and so we have added extensive simulations to understand the behavior of `FEEMS` and `FEEMSmix` in these scenarios. We include our findings in the Discussion in lines 712-729 (also see Fig S18-S20). Briefly, with strongly biased sampling, a scheme in which an entire half of the habitat is not sampled, the method fails completely (as expected under any reasonable assessment of what might be possible with limited data, see Fig S19). However, with a soft bias, where only 10 samples are included in this previously unsampled area, `FEEMSmix` picks up the direction and strength of long-range gene flow accurately in all replicates, though the migration surface is still quite poorly estimated (see Fig S20). This is now included in the Discussion section with practical recommendations for sampling.

If the authors see it fit, exploring the behavior of FEEMSmix under a panmictic model would also be a nice addition, illustrating the benefits of using this approach for testing the null hypothesis of no correlation between genetic and physical distances.

This is a valid point and we have implemented it. We note that it is slightly out of scope as `EEMS/FEEMS` is meant to help understand geographic structure when there is evidence of such geographic structure to begin with (e.g., the slope between measures of pairwise geographic and genetic distance is positive or visual patterns of geographic structure in a PCA plot with points colored by geographic sampling locations or population labels). If a population does not show any geographic structure with basic queries it is not advised to run `EEMS/FEEMS` or `FEEMSmix`. However, as we were also interested, we simulated under panmixia to show how `FEEMS` and `FEEMSmix` would perform. Though on running `FEEMS`, we observe flat migration surfaces estimated across all simulation replicates in accord with their being no spatial heterogeneity in geographic structure – and we find statistical support for some long-range edges as might be expected given panmixia involves long-range migration everywhere. The distribution of the slope between geographic and genetic distance is centered around zero (Fig S12), which should signal to users that attempting to model details of geographic structure with `FEEMSmix` is not advisable . We now emphasize this in the Discussion (lines 668-673).

Likelihood of 'FEEMSmix' model

Although the description of the algorithm which FEEMSmix relies on gives a fair overview of the underlying rationale, specific details are difficult to grasp. For instance, in Equation (1), I could not understand the second line (it should read $(1-c)T_{sd} + cT_{ss}$, I think). More importantly, the way values of Δ'_{ij} are calculated is very difficult to understand. In particular, it is not clear what R_{is}, R_{sj}, and $\hat{q}_s$ correspond to (I could not find their definitions elsewhere in the manuscript).

We apologize for the typo and lack of clarity — and we include a short paragraph following the equation array to explain the statistical model in further detail (in lines 296-300, lines 317-324 and lines 331-345). We hope that this addresses the concern.

Miscellaneous comments, questions and remarks

lines 165-172: What is the rationale for using a threshold of 2 log-likelihood units for testing the directionality of long-range migration events? Is it related to a quantile of a Chi-square distribution? Should that threshold vary as a function of the sample size?

Yes, exactly. We use 2 log-likelihood units as this represents a 95% quantile for a chi-squared distribution with a single degree of freedom. This is the appropriate distribution to use under the likelihood ratio test (LRT, Wilks 1938) with one extra parameter over the baseline (here, the source fraction for the single LRE over the estimated migration surface). Because the LRT already scales with sample size (larger datasets produce larger likelihood differences for the same effect size), no additional sample size adjustment is needed.

lines 310-318: The leave-one-out cross-validation procedure conducted here is quite interesting but the obtained results are described too briefly, in a vague manner unfortunately.

We have now given this section a separate subheading and added new sentences (lines 548-561) to explain the procedure in more detail.

Figure 5: the difference of R^2 with and without LRE seems very small here (compared to that observed for the simulations). Is there any way one could formally test that adding LREs leads to a statistically significant increase of the log-likelihood?

This is a valid point, and we have attempted to improve the methodology in this respect (see our response to the editor above).

Line 556: remove "not"?

Good catch, this has been corrected.

lines 575-578: what is the motivation for reformulating the expected covariance matrix the way that the authors did?

We do not technically reformulate the expected covariance matrix here, but simply convert the distance matrix to a covariance matrix. This is a common transformation used in statistical literature and is called the *centering method* (Gower 1966). This citation is now included in the main text. The reason we have to do this transformation is because the `FEEMS` gradient optimization machinery is built for a genetic covariance matrix, but the pairwise coalescent times are directly related to distance matrix (as modeled in `EEMS`). This is stated in lines 302-309 and lines 397-405 of the main text.
